# Cytokine trajectories after cardiopulmonary bypass in children: a systematic review and quantitative synthesis

**DOI:** 10.1016/j.ijcchd.2026.100698

**Published:** 2026-07-25

**Authors:** Andrea Elizabeth Pisesky, Colm Breatnach, Farid Foroutan, Christopher Parshuram, Alejandro Floh

**Affiliations:** a Department of Pediatrics, Stollery Children's Hospital, University of Alberta, 8440 112 St NW, Edmonton, T6G 2B7, Canada; b Intensive Care Medicine, Children's Health Ireland at Crumlin, Cooley St, Dublin, D12 N512, Ireland; c Ted Rogers Centre for Heart Research, University Health Network, Toronto, Canada; d Department of Critical Care Medicine, The Hospital for Sick Children, 170 Elizabeth Street, Toronto, M5G 1E8, Canada; e Centre for Safety Research, Child Health Evaluative Sciences, The Research Institute, The Hospital for Sick Children, 170 Elizabeth Street, Toronto, M5G 1E8, Canada; f Department of Paediatrics and Interdepartmental Division of Critical Care Medicine, University of Toronto, 27 King's College Circle, Toronto, M5S 1A1, Canada; g Labatt Family Heart Centre, Division of Cardiac Critical Care, The Hospital for Sick Children, Toronto, M5G 1E8, Canada

**Keywords:** Pediatric cardiac surgery, Cardiopulmonary bypass, Cytokine kinetics, Postoperative inflammation

**Figure:**
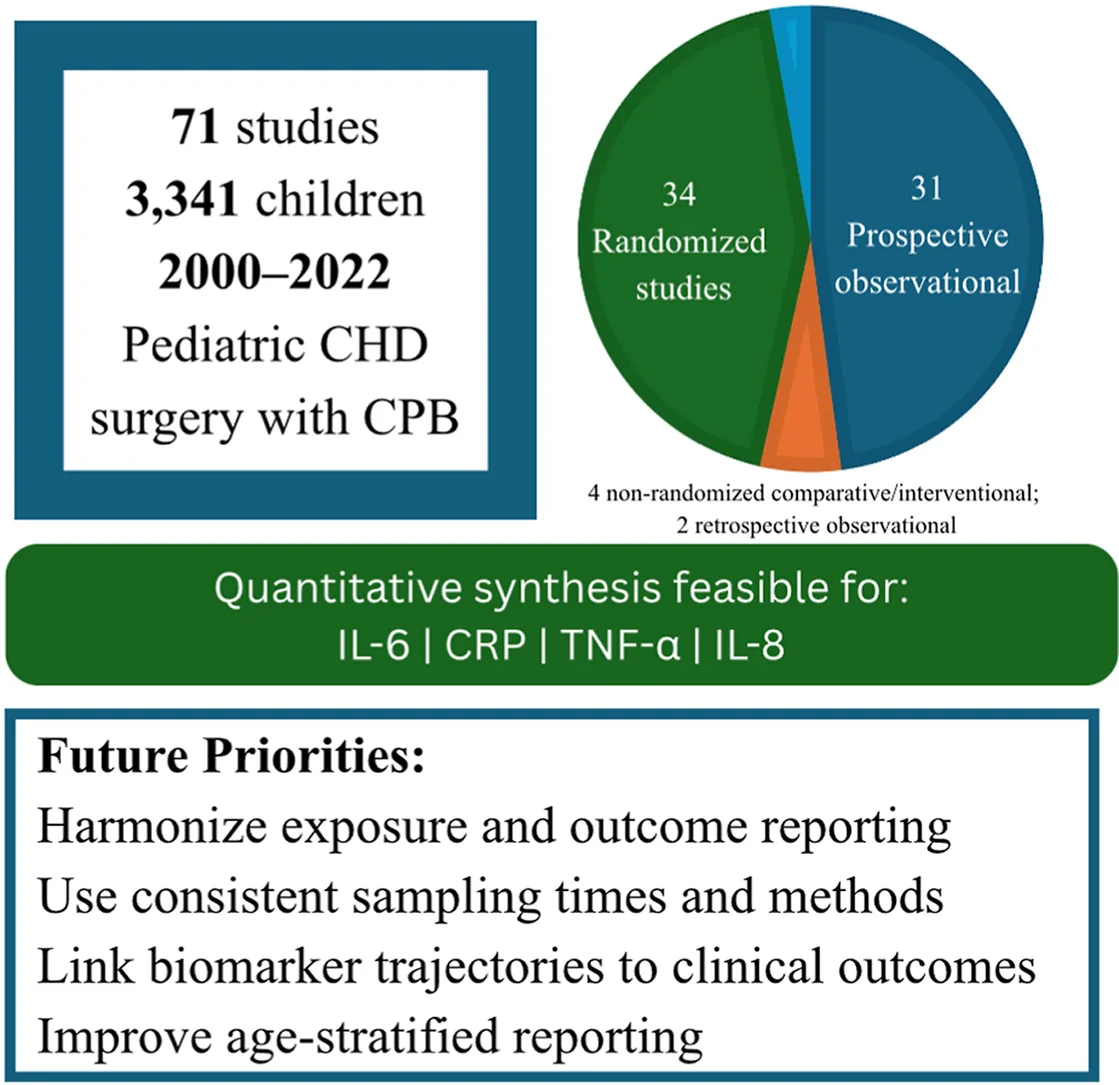

## Glossary of abbreviations

CPB: cardiopulmonary bypass
CRP: c-reactive protein
ICU: Intensive Care Unit
IL: interleukin
IQR: interquartile range
TNF-α: Tumor Necrosis Factor Alpha

## 1 Introduction

Cardiopulmonary bypass (CPB) provokes a systemic inflammatory response with important consequences for infants and children undergoing repair of congenital heart disease [[1], [2], [3]]. Inflammation engages overlapping coagulation and immune cascades that can culminate in endothelial dysfunction, vasoplegia, and organ injury, contributing to postoperative morbidity and delayed recovery in a substantial subset of patients [[4], [5], [6], [7], [8], [9]].

Efforts to blunt the postoperative inflammatory response in children have largely used global modulation strategies—biocompatible, heparin-coated circuits, nitric oxide delivered into the circulation, perioperative corticosteroids, and modified ultrafiltration—but clinical benefits have been inconsistent [[10], [11], [12], [13], [14]]. By contrast, targeted cytokine therapies in other hyperinflammatory conditions—tocilizumab (Interleukin (IL)-6 receptor blockade) [15], anakinra (IL-1 receptor antagonist) [16], and infliximab or etanercept (Tumor necrosis factor alpha (TNF-α) inhibitors) [17]—show clear clinical utility. These successes highlight the promise of precision immunomodulation in pediatric cardiac surgery, if relevant pathways are delineated.

We therefore conducted a systematic review to synthesize cytokine measurements after pediatric cardiopulmonary bypass, characterize temporal patterns across prespecified postoperative windows, and evaluate certainty of evidence. Our aim was to identify reliable postoperative signals—particularly potential anchor biomarkers—that could inform standardized measurement, link profiles to outcomes, and enable rational design and timing of mechanism-based anti-inflammatory trials.

## 2 Methods

We registered the protocol (PROSPERO CRD42022238950) and adhered to PRISMA 2020. We included original studies enrolling patients younger than 18 years undergoing CPB for repair of congenital heart disease that reported at least one circulating cytokine or inflammatory biomarker. We excluded adult-only cohorts, animal or in vitro studies, conference abstracts, editorials, and narrative reviews. Only full-text, English-language articles published from January 1, 2000, through January 1, 2022 were considered.

**Fig. 1:**
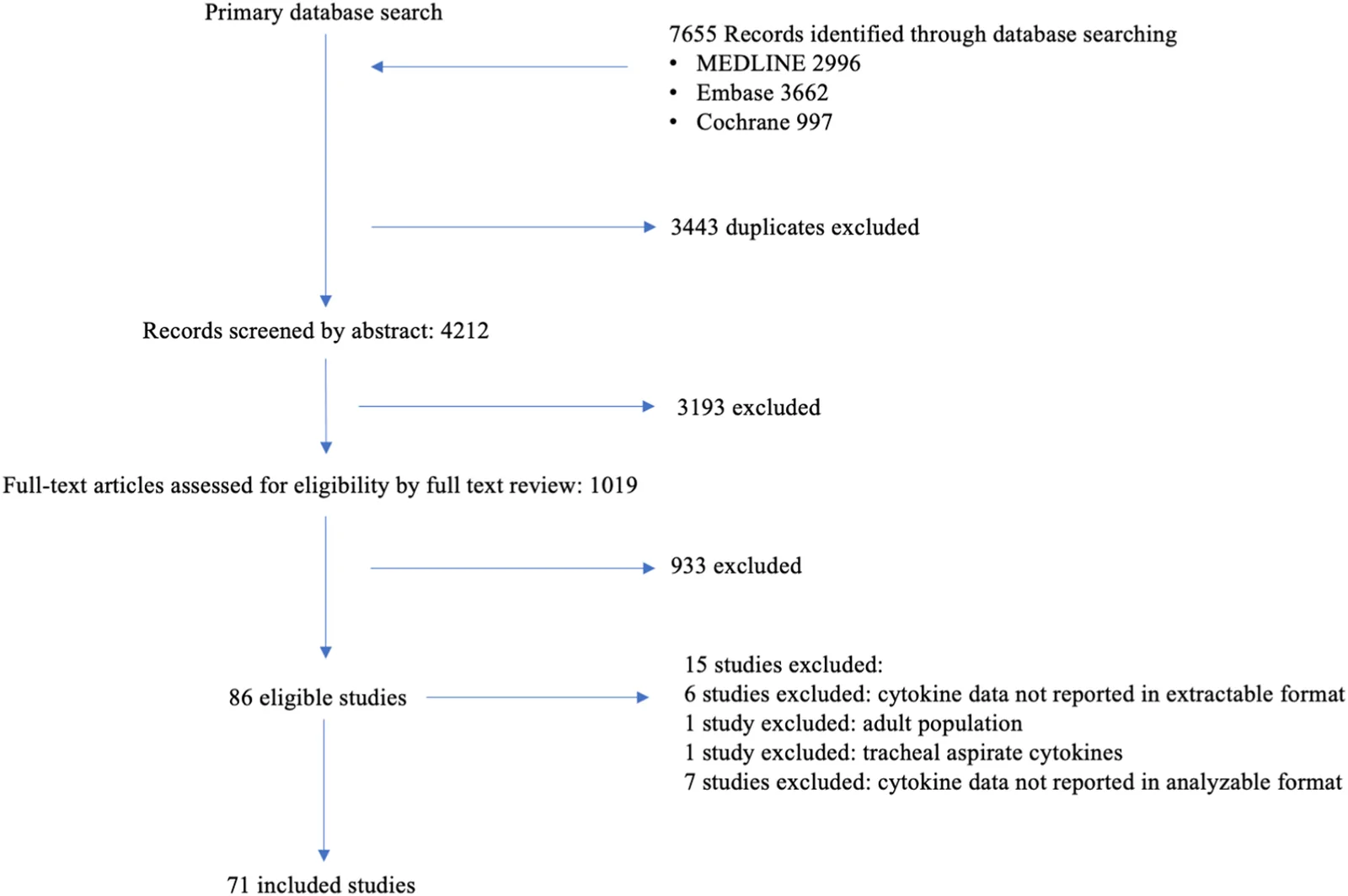
PRISMA 2020 flow diagram illustrating the study selection process. Legend: A total of 7655 records were identified through the primary database search. After removal of 3443 duplicates, 4212 records underwent title and abstract screening. Of these, 1019 full-text articles were assessed for eligibility. Following initial full-text review, 933 studies were excluded. Eighty-six studies were retained for detailed evaluation, from which an additional 15 were excluded due to lack of analyzable cytokine data or ineligible study populations. Seventy-one studies were included in the final synthesis.

An information specialist designed and executed electronic searches in MEDLINE (Ovid), Embase (Ovid), the Cochrane Central Register of Controlled Trials, and the Cochrane Database of Systematic Reviews. Searches were structured around two concepts: cardiopulmonary bypass/cardiac surgery and cytokines/inflammation. Strategies combined controlled vocabulary and free-text terms within each concept, and records were retrieved when they included at least one term from each domain. The search was limited to English-language full-text articles published from January 1, 2000, to January 1, 2022. Full database-specific search strategies are provided in Supplemental text, Appendix 1. We also screened reference lists of all eligible full-text articles. Two reviewers (A.P., C.R.B.) independently screened titles/abstracts and full texts; disagreements were adjudicated by a third (A.F.). Full-text exclusion reasons are shown in the PRISMA flow diagram (Fig. 1).

Data were extracted independently in duplicate using a standardized form by the same reviewer pairs. We recorded study/participant characteristics, bypass variables (duration, cross-clamp time, deep hypothermic circulatory arrest), perioperative interventions, cytokine assay details and sampling windows, and clinical outcomes (mortality, ventilation duration, Intensive Care Unit (ICU) and hospital stay, complications). When only medians and interquartile ranges (IQRs) were reported, IQR was converted to SD (IQR÷1.35). Discrepancies were resolved by consensus.

Biomarker units were standardized (C-reactive protein (CRP), mg/dL; all others, pg/mL). Values were log-transformed for pooling and back-transformed to the original scale. Sampling schedules varied across studies. We therefore grouped measurements into pragmatic perioperative time windows based on the most frequently reported sampling anchors: baseline; immediately post-bypass; 0.5–2 h, 3–4 h, 6–8 h, 12 h, 18–20 h; postoperative day 1, day 2, and day 3. These windows were intended to preserve clinically interpretable phases of the perioperative course while enabling synthesis across heterogeneous reporting schedules, rather than to define biologically discrete inflammatory phases.

For cytokine–timepoint cells with ≥3 cohorts, we pooled log-transformed means using random-effects, inverse-variance weighting (DerSimonian–Laird) and back-transformed results. When interventional studies reported arms separately, both arms were included. For interventional studies, arms were retained separately and analyzed to avoid double-counting shared participants (adjusted variances per Cochrane guidance). Heterogeneity was assessed with I [2]. We produced forest plots by time window and a **c**omposite trajectory from pooled estimates. All analyses were conducted in R version 4.3.2.

For each biomarker, forest plots were generated separately for each analyzable perioperative time window, displaying individual contributing cohort estimates and the corresponding random-effects pooled estimate. Because each time window represented a distinct synthesis with different contributing cohorts and sampling anchors, estimates from different time windows were not combined into a single forest plot.

Certainty of evidence for each cytokine–timepoint synthesis was rated using the GRADE framework, considering risk of bias, inconsistency, indirectness, imprecision, and publication bias. Because this review synthesized descriptive biomarker concentrations rather than intervention effects, GRADE ratings reflected certainty in the reported temporal biomarker pattern and pooled concentration estimate at each time point. Ratings therefore considered not only study design, but also the number of contributing cohorts, width of confidence intervals, between-study heterogeneity, assay and sampling variability, and generalizability of the absolute pooled estimate.

## 3 Results

**Fig. 2:**
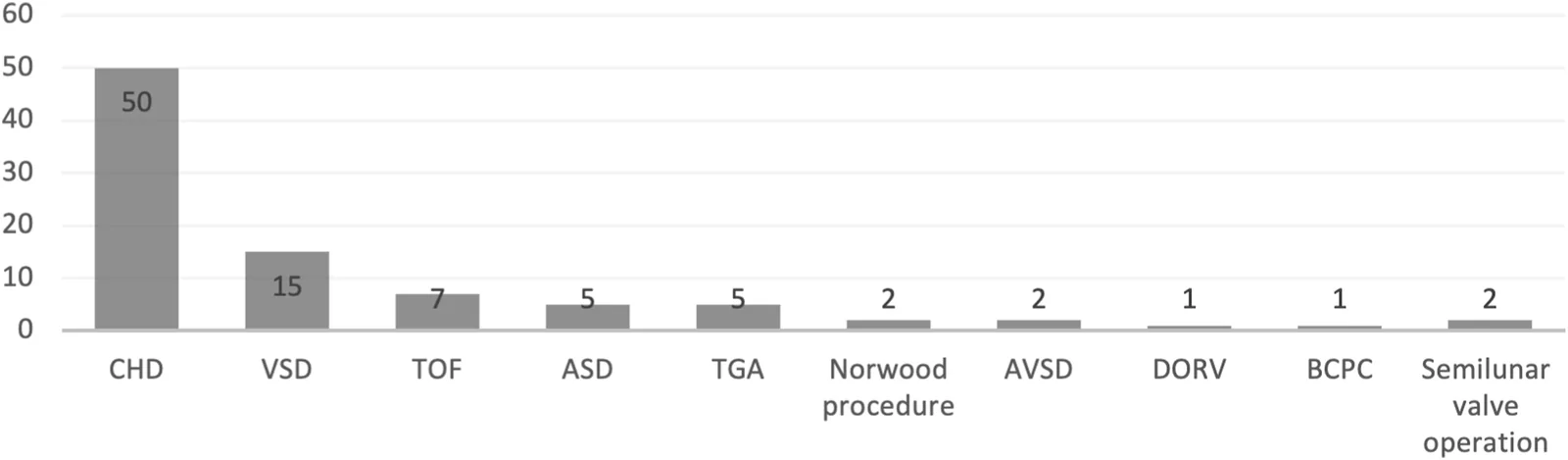
Number of studies including patients with the following cardiac diagnoses or surgical procedures. Abbreviations: ASD – atrial septal defect; AVSD – atrioventricular septal defect; BCPC – bidirectional cavopulmonary connection; CHD – congenital heart disease; DORV – double outlet right ventricle; TOF – Tetralogy of Fallot; VSD – ventricular septal defect.

A total of 7655 records were identified; 71 pediatric studies met inclusion (Fig. 1). Among the 71 unique included studies enrolling 3341 patients (range, 9–168 patients per study; Supplemental text, Table 1), 34 (47.9%) were randomized interventional studies, 4 (5.6%) were non-randomized interventional or comparative studies, 31 (43.7%) were prospective observational or descriptive studies, and 2 (2.8%) were retrospective observational studies. Seventy of seventy-one (99%) reported age and 65/71 (92%) reported a weight measure (body weight, body surface area, or body mass index) at the index operation (Supplemental Text, 2). Study-level age eligibility varied across the 71 unique included studies. Five studies (7%) were neonate-focused, defined as enrolling newborns/neonates ≤28–30 days; 15 (21%) were infant-focused, enrolling children within the first year of life; 18 (25%) enrolled young child or weight-based cohorts, in which eligibility crossed the 12-month boundary or was defined by low body weight; 17 (24%) enrolled broad pediatric cohorts spanning infancy through childhood; and 5 (7%) were older child-focused, enrolling children >12 months or excluding infants. Three studies (4%) explicitly compared age groups or reported age-stratified findings, and 8 (11%) had unclear or inconsistent age eligibility. Although neonates and infants were represented, age-specific synthesis was limited by mixed populations and inconsistent age-stratified biomarker reporting. Twenty-one (30%) studies had a relatively homogeneous case mix (≤3 cardiovascular diagnoses/procedures) (Fig. 2).

Among 34 randomized interventional trials, interventions targeted anesthetic management (9, 13%), bypass techniques (6, 8%), preoperative steroids (6, 8%), and ultrafiltration (5, 7%; Supplemental text, Table 2). Perioperative steroids were used in 21/71 (30%) studies.

Mortality was reported in 22/71 (31%) studies; 39/1345 (3%) patients died. ICU length of stay was reported in 38/71 (54%), duration of ventilation in 46/71 (65%), and hospital length of stay in 16/71 (23%) studies. Other complications (acute kidney injury, sepsis, wound dehiscence, oxygenation index, sternal re-opening) were infrequently reported (Supplemental Text, Table 3).

**Cytokine Results Overview.** Studies reported a limited set of cytokines—CRP, interferon-γ, interleukin-10, interleukin-18, interleukin-1β, interleukin-6, interleukin-8, monocyte chemoattractant protein-1, and tumor necrosis factor α (Supplemental Text, Table 3). Quantitative analyses were feasible for CRP, IL-6, IL-8, and TNF-α (≥3 studies). Individual studies measured a median of 2 cytokines (range, 1–28) over 4 time points (range, 1–13). Baseline values were provided in 66/71 (93%) studies: 9 preoperative, 51 intraoperative near induction, 2 at bypass initiation, and 14 with unspecified timing. Measurements while on bypass/ultrafiltration occurred in 7/71 (10%). Postoperative sampling was anchored to separation from bypass or protamine in 55 (77%), anesthesia induction in 1 (1%), skin incision in 1 (1%), and ICU admission in 14 (20%). Only 7 (10%) studies extended beyond postoperative day 3.

Across biomarkers and sampling windows, pooled estimates were accompanied by wide confidence intervals, particularly for early postoperative IL-6 and TNF-α and for later time points with few contributing cohorts. These wide intervals reflect substantial between-study heterogeneity, including differences in age distribution, case mix, CPB duration, perioperative steroid and ultrafiltration exposure, assay platforms, sampling anchors, and reporting formats. Accordingly, pooled estimates should be interpreted as descriptive summaries of reported biomarker concentrations within broad postoperative windows rather than precise reference values or clinically actionable thresholds.

**Fig. 3:**
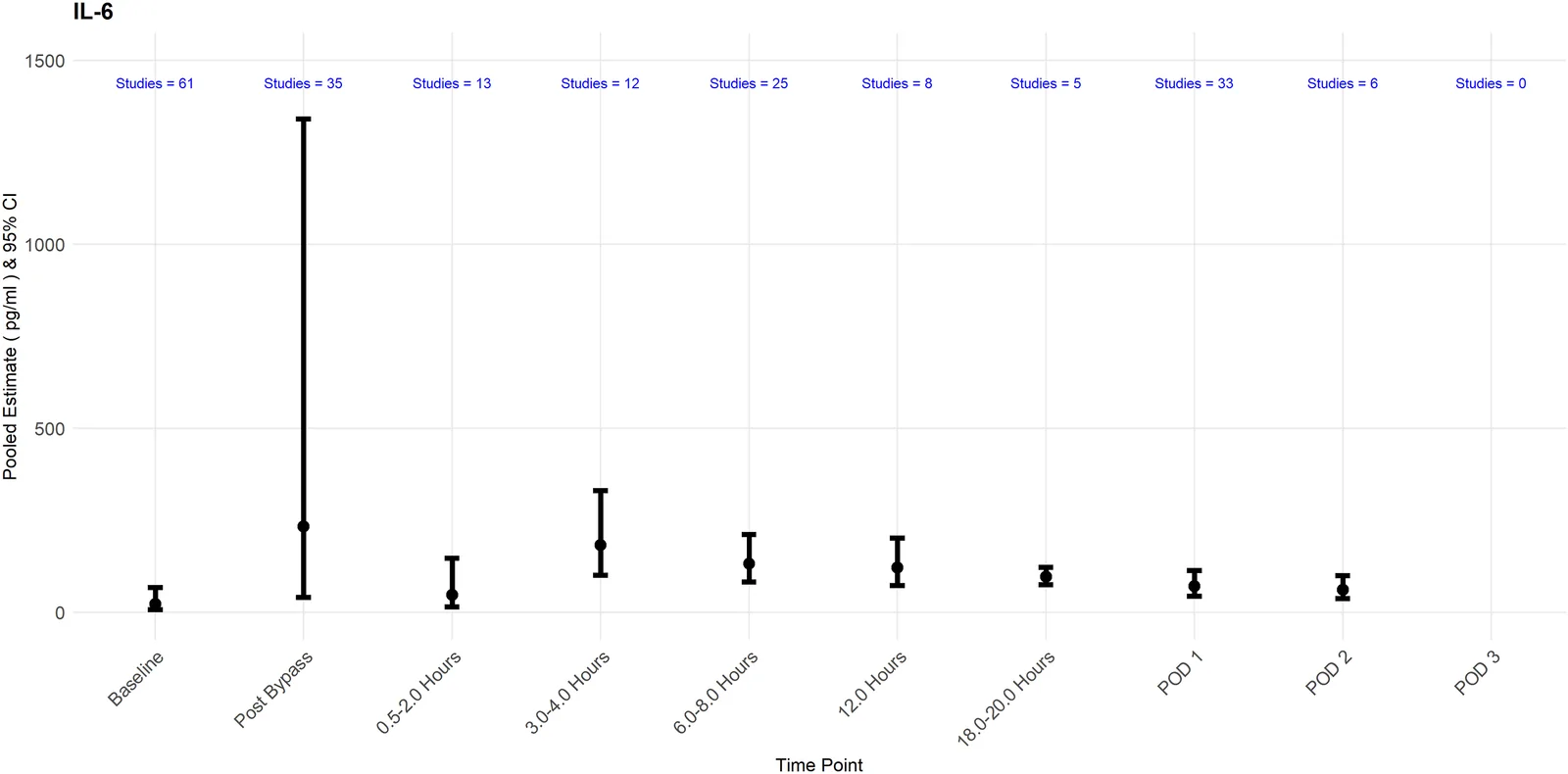
Pooled interleukin-6 (IL-6) concentrations following cardiopulmonary bypass at sequential postoperative time points.

**Interleukin-6.** Thirty-three studies (46%) reported IL-6 (63 cohorts, Fig. 3). The pooled estimate of baseline IL-6 was 22.40 pg/ml (95% CI 7.37, 68.05 pg/ml), rising immediately post CPB peaked at 233.02 pg/ml (95% CI 40.49, 1340.98 pg/ml) in 35 cohorts. The wide confidence interval around the immediate post-CPB estimate indicates substantial variability across cohorts and limits interpretation of the absolute pooled concentration, although the direction of early postoperative increase was consistent. Thirty to 120 min following CPB, the value initially declined to 47.40 pg/ml (95% CI 15.26, 147.23 pg/ml) and peaked at 3-4 h post CPB to 182.65 pg/ml (95% CI 100.94, 330.50 pg/ml) in 25 cohorts. The final nadir was on post operative day 2 at 61.55 pg/ml (95% CI 37.94, 99.85 pg/ml). No study had analyzable IL-6 data beyond the second post operative day. One ultrafiltration trial (Wang et al. [18]) was a clear outlier at baseline and immediately post CPB.

**Fig. 4:**
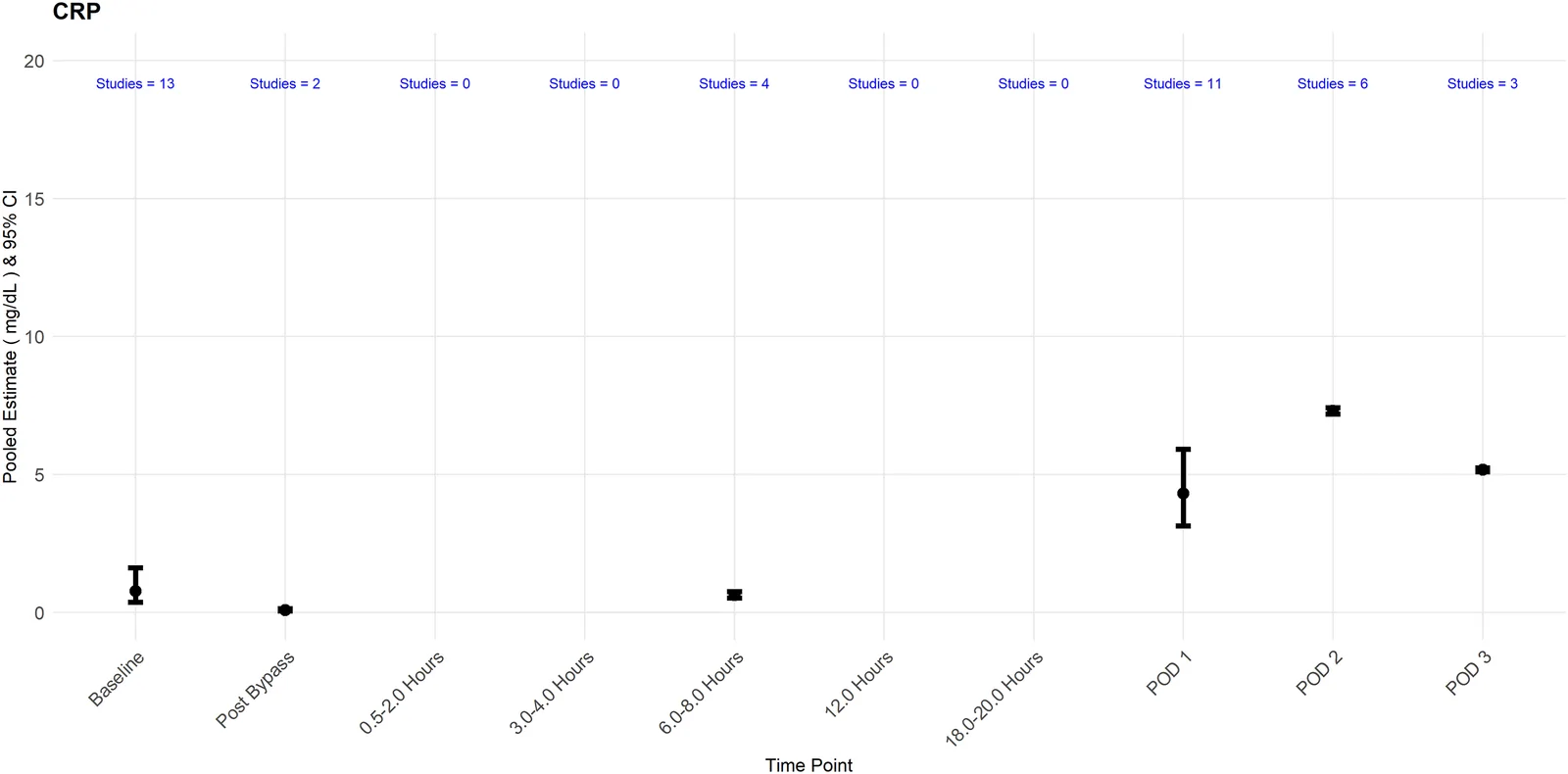
Pooled C Reactive Protein (CRP) concentrations following cardiopulmonary bypass at sequential postoperative time points.

**C-Reactive Protein.** Sixteen (23%) studies (19 cohorts) reported CRP (Fig. 4). The pooled estimate before bypass was 0.77 mg/dL (95% CI 0.37, 1.62); immediately after bypass it was 0.09 mg/dL (95% CI 0.05, 0.15). A peak occurred on postoperative day 2 (7.3 mg/dL [95% CI 7.19, 7.42]; six cohorts), and values remained above baseline on postoperative day 3 (5.17 mg/dL [5.10–5.24]). No analyzable data were available at 0.5-2 h, 3–4 h, 12 h, or 18–24 h.

**Fig. 5:**
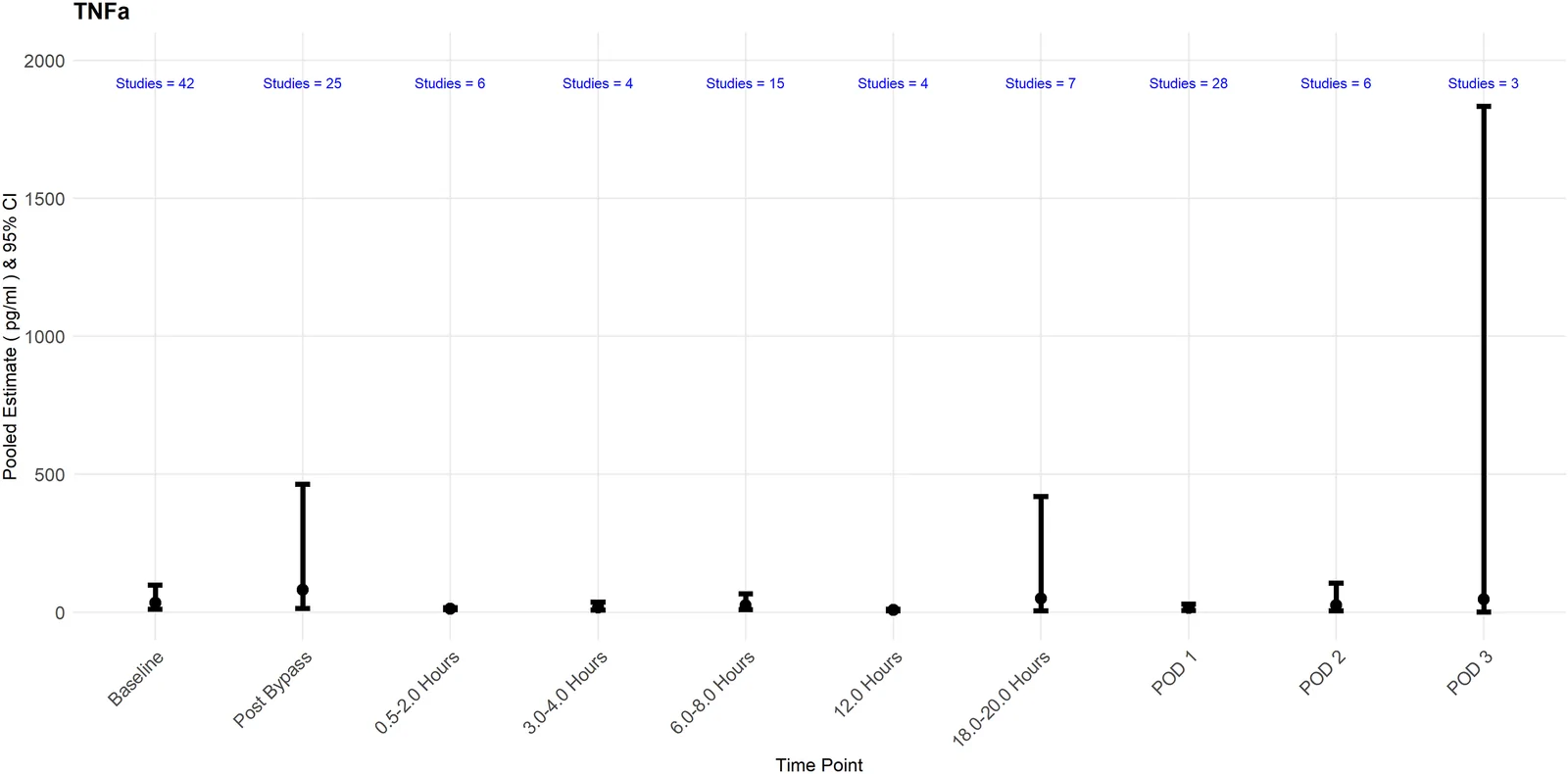
Pooled Tumor Necrosis Factor Alpha (TNF- α) concentrations following cardiopulmonary bypass at sequential postoperative time points.

**Tumor Necrosis Factor α.** Fourteen (20%) studies (42 cohorts) reported TNF-α (Fig. 5). The pooled estimate before bypass was 33.95 pg/ml (95% CI 11.62, 99.15); immediately after bypass it was 81.72 pg/ml (95% CI 14.39, 464.13; 25 cohorts). The nadir at 12 h was 8.23 pg/ml (95% CI 5.62, 12.05). Levels rose again at 18–20 h to 50.88 pg/ml (95% CI 6.17, 419.74) and continued to increase by postoperative day 3 to **46.99** pg/ml (95% CI 1.20, 1834.3; three cohorts). Estimates at later time points were especially imprecise, and the postoperative day 3 estimate was based on only three cohorts; this late apparent rise should therefore be interpreted cautiously. No analyzable data were available beyond postoperative day 3.

**Fig. 6:**
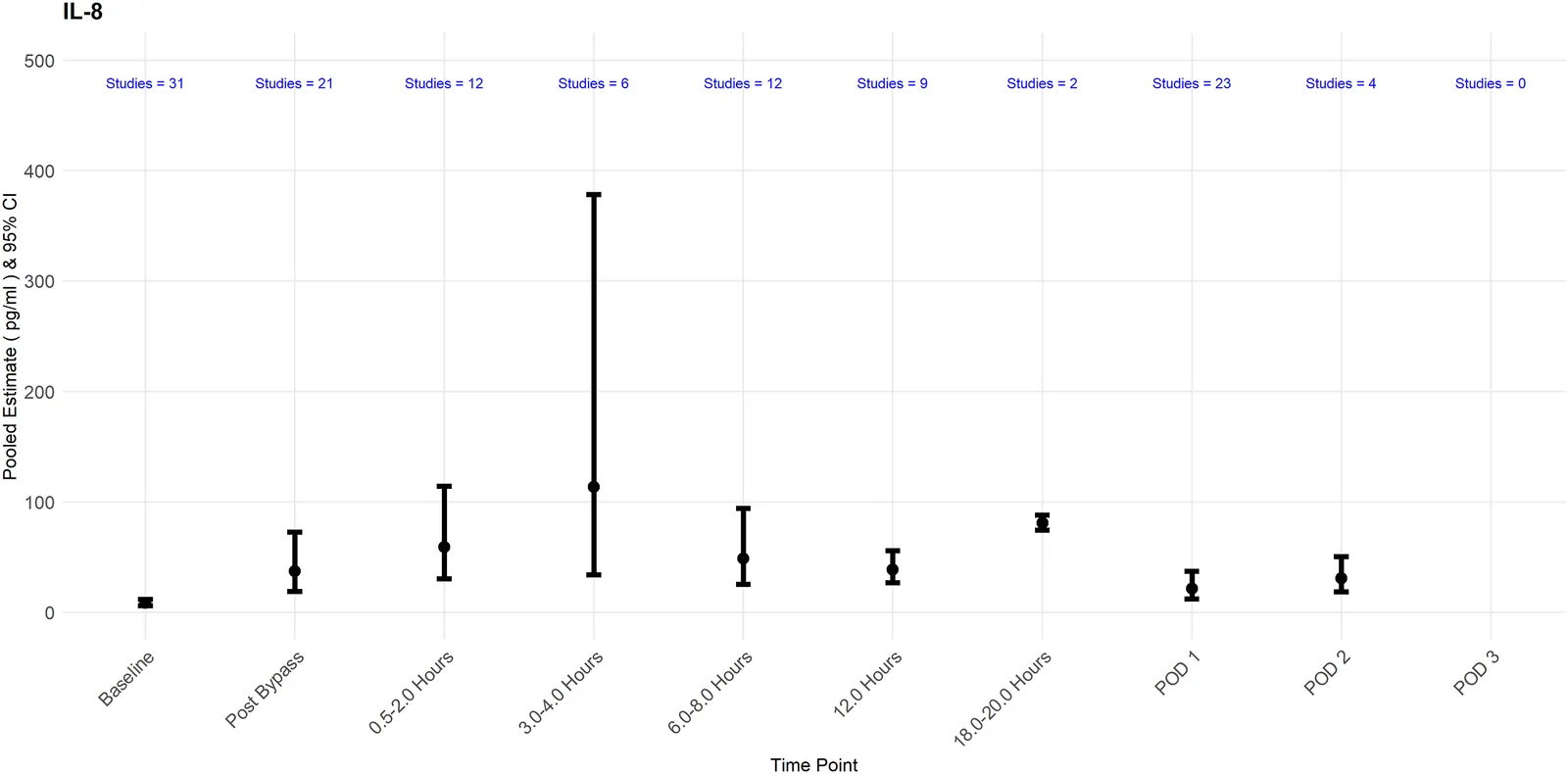
Pooled Interleukin-8 (IL-8) concentrations following cardiopulmonary bypass at sequential postoperative time points.

**Interleukin-8.** Eight (11%) studies (31 subgroups) reported IL-8 (Fig. 6). The pooled pre-bypass estimate was 8.54 pg/ml (95% CI 6.09, 11.99). Levels peaked at 3–4 h post-bypass at 113.59 pg/ml (95% CI 34.09, 378.44; six cohorts) and showed a second rise at 18–20 h to 81.12 pg/ml (95% CI 74.59, 88.22; two cohorts). By postoperative day 2, values remained above baseline at 30.72 pg/ml (95% CI 18.62, 50.68). No analyzable data were available beyond postoperative day 2.

**Risk of Bias.** Risk of bias varied by study design. Randomized interventional studies were most commonly limited by incomplete reporting of allocation concealment, blinding, or selective cytokine outcome reporting. Non-randomized and observational studies were mainly limited by confounding, heterogeneous case mix, and incomplete adjustment for perioperative factors including age, diagnosis, CPB duration, steroid exposure, ultrafiltration, and assay platform. Across study designs, the most frequent concern was selective or incomplete biomarker reporting. A summary of risk-of-bias assessments is provided in Supplemental Text, Table 5; full study-level assessments are available on request.

**Certainty of Evidence (GRADE).** Certainty of evidence varied by biomarker and time point. For IL-6, certainty was moderate at better-supported postoperative windows and low where estimates were based on fewer contributing cohorts, mixed study designs, wide confidence intervals, or substantial heterogeneity. Although IL-6 showed a recurring pattern of early postoperative elevation followed by decline, certainty in the absolute pooled concentrations was limited by heterogeneity in patient populations, perioperative exposures, assay platforms, sampling schedules, and reporting. For CRP, certainty was low or very low across available time points because data were sparse and several perioperative windows had no contributing cohorts. For IL-8, certainty was moderate at selected better-supported windows but low or very low where data were sparse, mixed-design, or imprecise. For TNF-α, certainty was low or very low across most time points because of wide confidence intervals, heterogeneity, and limited reliability of later postoperative estimates. Overall, GRADE assessments support interpretation of pooled estimates as descriptive summaries of reported biomarker trajectories rather than precise reference values or clinically actionable thresholds (Supplemental Text, Fig. 1; Tables 6–9).

## 4 Discussion

In this systematic review and quantitative synthesis, we describe the inflammatory cytokine response in children undergoing cardiopulmonary bypass, synthesizing 71 studies and 3341 patients. Despite methodological variability and heterogeneous case mix, recurring patterns emerged that clarify the temporal dynamics of postoperative inflammation in this population and highlight the need for standardized outcome-linked biomarker studies.

**Time to Peak Cytokine Levels.** The early postoperative cytokine response appears to be dominated by pleiotropic mediators, particularly IL-6 and TNF-α. Among all cytokines, IL-6 showed the most consistently reported pattern—an early postoperative rise with a secondary elevation at 3–4 h and decline by postoperative day 2. However, certainty was not uniformly high across IL-6 time points. Several estimates were supported by RCT-derived cohorts, but other windows included fewer contributing cohorts, mixed study designs, wide confidence intervals, or substantial heterogeneity. These findings support IL-6 as a useful descriptive anchor for postoperative inflammatory profiling, but not as a precise reference concentration, prognostic threshold, or treatment-selection marker. One ultrafiltration trial (Wang et al.) exaggerated the earliest peak, underscoring the need for standardized sampling windows and transparent perioperative reporting [18]. Biologically, interleukin-6 is produced by monocytes/macrophages, T cells, fibroblasts, and endothelium and exerts both pro- and anti-inflammatory effects via activation of the JAK/STAT and MAPK signaling pathways; the observed lag in CRP aligns with IL-6–induced hepatic synthesis via STAT3 activation with this pathway [19]. The robustness of IL-6 also makes it a practical anchor for genetics-informed work. Adult cardiac surgery cohorts have linked IL-6 promoter variation (e.g., −174 G/C) to higher postoperative interleukin-6 concentrations and worse clinical trajectories [20,21]; pediatric data are sparse but directionally consistent [22]. These signals support future studies testing whether host IL-6 genotype contributes to between-patient differences in IL-6 profiles and whether these profiles are associated with postoperative outcomes.

Our synthesis supports TNF-α as an early mediator after cardiopulmonary bypass: concentrations peak immediately post-bypass and return toward baseline within 30–120 min, consistent with release during tissue injury and ischaemia–reperfusion [23]. Prior work shows similar early elevation during myocardial reperfusion and, to a lesser extent, after off-pump surgery, implicating reperfusion rather than the extracorporeal circuit alone [[24], [25], [26]]. Biologically, TNF-α —produced predominantly by monocytes/macrophages—activates endothelium, recruits leukocytes, and amplifies inflammation via NF-κB [27], aligning with the observed early but lower-certainty signal in our pooled data.

Lastly, IL-8 increased postoperatively with a 3–4 h peak, consistent with endothelial activation and neutrophil recruitment that may drive microvascular injury [[28], [29], [30], [31]]. However, measurement was less consistent and late sampling sparse, yielding lower certainty than for IL-6. Exploratory adult data suggest that IL-8 promoter variants (e.g., −251 A/T) and chemokine-receptor polymorphisms (CXCR1/CXCR2) influence IL-8 production and neutrophil responses, but pediatric cardiac-surgery evidence is limited [[32], [33], [34], [35]]. Accordingly, IL-8 is best treated as a supportive, neutrophil-linked marker within profiles anchored on IL-6.

**Biphasic Inflammatory Patterns.** Several cohorts suggested secondary rises of TNF-α and IL-8 at 18–72 h after CPB. While the 3–4 h IL-8 peak likely reflects acute innate activation, the later rise (∼18–24 h) is biologically plausible as a second hit from ongoing endothelial activation, delayed DAMP release, and neutrophil re-recruitment/NETosis (including IL-8 release from Weibel–Palade bodies) [8,31,36,37]. C-reactive protein, hepatically induced downstream of IL-6/STAT3, has slower kinetics (production lag, ∼19-h half-life) and thus integrates upstream signals; its persistence to postoperative day 3 likely reflects ongoing IL-6 activity/tissue injury [19]. Interpretation is limited by sparse, heterogeneous late sampling and nonspecific perioperative influences. Overall, the data fit a multiphasic model but remain hypothesis-generating; standardized sampling beyond 72 h is needed to determine prognostic and therapeutic relevance.

**Limitations**. We rated certainty with GRADE for the four most reported markers (IL-6, CRP, IL-8, and TNF-α) across prespecified time points. Certainty varied by biomarker and sampling window and was influenced by the number and design of contributing cohorts, confidence interval width, between-study heterogeneity, assay and sampling variability, and generalizability of the absolute pooled estimates. IL-6 provided the most consistently reported descriptive trajectory, with recurrent early postoperative elevation and subsequent decline, but certainty was moderate only at selected better-supported windows and low where data were sparse, mixed-design, imprecise, or heterogeneous. CRP and IL-8 showed low to moderate certainty at selected time points, while TNF-α was generally low or very low owing to heterogeneity, imprecision, and unstable late estimates. These findings underscore the need for standardized sampling, transparent assay reporting, and prospective linkage between biomarker trajectories and clinical outcomes.

Reporting of assay performance was infrequent; few studies detailed reliability, limits of detection, or intra/inter-assay variability, limiting confidence in concentration estimates and between-study comparisons. It was also unclear whether cytokine panels were comprehensive or selectively reported based on study design or assay availability. Universally applicable pediatric reference ranges were not applied because biomarker concentrations vary by assay platform, units, sample type, age, and laboratory. Preoperative or baseline values therefore provide the most relevant internal comparator, and postoperative estimates should be interpreted in relation to baseline values and temporal patterns rather than a single normal/abnormal threshold.

One outlier in the tumor necrosis factor α synthesis was Talwar et al. a randomized trial comparing Del Nido vs St. Thomas cardioplegia. Although between-group differences were absent, elevated levels overall raise the possibility that cardioplegia technique or myocardial reperfusion contributed. Given limited methodological detail—especially on sampling protocols and assay methods—it remains uncertain whether these elevations reflect biology or technical variability [38].

We limited inclusion to studies reporting absolute cytokine concentrations; studies presenting only relative changes or alternative inflammatory markers were excluded. We also included interventional trials (e.g., ultrafiltration, corticosteroids) when cytokine data were extractable and the intervention showed no statistically significant effect (i.e., non-superior/neutral), analysing them alongside observational cohorts. Variability in how these interventional cohorts were handled and reported may have influenced pooled estimates.

Most included studies were observational with limited control for key confounders, constraining causal inference. Reporting of clinical outcomes—ICU length of stay, duration of mechanical ventilation, and postoperative complications—was inconsistent, and important morbidities linked to inflammation (e.g., acute kidney injury, prolonged ventilation, sepsis) were rarely captured systematically. These limitations hinder a cohesive understanding of postoperative inflammatory trajectories in pediatric cardiopulmonary bypass populations [3,39].

Future work should focus on large, multicentre studies using standardized sampling windows anchored to surgery and cardiopulmonary bypass. Cytokine quantification must address pre-analytic variables (timing, handling, assay choice). Surgical factors (cardioplegia strategy, ischaemia–reperfusion management) should be specified. Studies should perform serial, extended sampling beyond 72 h to capture possible secondary waves and define therapeutic windows, while prospectively linking biomarker trajectories to standardized patient- and clinician-important outcomes. These should include harmonized measures of postoperative morbidity, such as duration of mechanical ventilation, ICU and hospital length of stay, acute kidney injury, low cardiac output syndrome, infection, mortality, and recovery trajectories. In parallel, pair serial cytokine profiling with host inflammatory-pathway genotyping/omics (candidate cytokine/chemokine and receptor variants, plus genome-wide approaches with ancestry adjustment) to define inflammatory endotypes, improve risk stratification, and enable pharmacogenetic trials of targeted anti-inflammatory therapies.

## 5 Conclusion

This systematic review underscores the complexity of postoperative inflammation in children undergoing cardiopulmonary bypass and identifies recurring biomarker trajectories that may inform future outcome-linked studies. Interleukin-6 provides the most consistent postoperative signal, whereas tumor necrosis factor α and interleukin-8 are less consistently measured, and late sampling is uncommon. As a result, trajectories suggest a possible multiphasic course, but evidence is limited by small samples, heterogeneous protocols, and follow-up rarely beyond 72 h. Future work should standardize cytokine measurement, extend serial sampling, and prospectively link biomarker trajectories to harmonized patient- and clinician-important outcomes, so that inflammatory phenotypes can be translated into mechanism-based, targeted therapies aimed at reducing postoperative morbidity.

## Disclosures

None.

## Funding

None.

## CRediT authorship contribution statement

**Andrea Elizabeth Pisesky:** Writing – review & editing, Writing – original draft, Visualization, Validation, Resources, Project administration, Methodology, Investigation, Data curation, Conceptualization. **Colm Breatnach:** Data curation, Conceptualization. **Farid Foroutan:** Writing – review & editing, Visualization, Validation, Methodology, Formal analysis. **Christopher Parshuram:** Writing – review & editing, Supervision, Methodology, Conceptualization. **Alejandro Floh:** Writing – review & editing, Validation, Supervision, Project administration, Methodology, Conceptualization.

## Declaration of competing interest

The authors declare that they have no known competing financial interests or personal relationships that could have appeared to influence the work reported in this paper.
