# Supplementary material for: Cytokine trajectories after cardiopulmonary bypass in children: a systematic review and quantitative synthesis
Source: Int J Cardiol Congenit Heart Dis. 2026 Jul 25;25:100698. doi: 10.1016/j.ijcchd.2026.100698 (PMC13545453; doi:10.1016/j.ijcchd.2026.100698)
Supplement: Supplementary file 1 [file mmc1.docx]

**Supplemental Text**

**Appendix 1. Search strategy**

The search strategy was developed with an information specialist and structured around two core concepts: **(**1) cardiopulmonary bypass/cardiac surgery terms and (2) cytokine/inflammation terms. Records were retrieved when they included at least one term from each concept. Searches were conducted in MEDLINE, Embase, the Cochrane Central Register of Controlled Trials, and the Cochrane Database of Systematic Reviews. The search was limited to English-language full-text articles published from January 1, 2000, to January 1, 2022. Reference lists of included studies were also screened. The review protocol was registered in PROSPERO: CRD42022238950.

Databases: MEDLINE via Ovid, Embase and Cochrane
Date searched: May 15, 2021
Search period: January 1, 2000, to January 1, 2022

1. exp Cardiopulmonary Bypass/
2. cardiopulmonary bypass.mp.
3. CPB.mp.
4. cardiac surg*.mp.
5. congenital heart disease.mp.
6. 1 or 2 or 3 or 4 or 5
7. exp Cytokines/
8. cytokine*.mp.
9. interleukin*.mp.
10. IL-6.mp.
11. IL-8.mp.
12. tumor necrosis factor.mp.
13. TNF-alpha.mp.
14. C-reactive protein.mp.
15. inflamm*.mp.
16. 7 or 8 or 9 or 10 or 11 or 12 or 13 or 14 or 15
17. 6 and 16
18. limit 17 to English language
19. limit 18 to yr="2000 - 2022"

Table 1. Overview of the study design and cohort description of the 71 included studies

| Study ID | Year | Country | Study design | Cohorts | Patients | Description |
| --- | --- | --- | --- | --- | --- | --- |
| 1 | 2020^1^ | Finland | RCT | 2 | 14 | Placebo |
|  |  |  |  |  | 15 | Steroid treatment |
| 2 | 2001^2^ | China | RCT | 4 | 20 | Control |
|  |  |  |  |  | 20 | BUF |
|  |  |  |  |  | 20 | MUF |
|  |  |  |  |  | 20 | BUF and MUF |
| 3 | 2008^3^ | China | RCT | 2 | 30 | Routine therapy |
|  |  |  |  |  | 30 | Tight glucose control |
| 4 | 2012^4^ | Japan | RCT | 2 | 13 | Control |
|  |  |  |  |  | 13 | Sivelestat therapy |
| 5 | 2021^5^ | Finland | RCT | 2 | 20 | Control |
|  |  |  |  |  | 20 | Steroid treatment |
| 6 | 2004^6^ | Switzerland | Prospective observational cohort | 1 | 40 | Observational study |
| 7 | 2007^7^ | USA | Prospective observational cohort | 1 | 24 | Observational study |
| 8 | 2017^8^ | Iran | RCT | 2 | 32 | Del Nido Cardioplegia |
|  |  |  |  |  | 27 | St Thomas Cardioplegia |
| 9 | 2006^9^ | Canada | RCT | 2 | 20 | Control |
|  |  |  |  |  | 17 | RIPC |
| 10 | 2006^10^ | Netherlands | RCT | 2 | 15 | Non coated CPB circuit |
|  |  |  |  |  | 13 | Phosphorylcholine-coated CPB circuit |
| 11 | 2021^11^ | China | RCT | 2 | 84 | Control |
|  |  |  |  |  | 84 | Steroid treatment |
| 12 | 2005^12^ | UK | RCT | 2 | 28 | Normothermia |
|  |  |  |  |  | 31 | Hypothermia |
| 13 | 2001^13^ | Spain | Prospective observational cohort | 2 | 8 | Neonates |
|  |  |  |  |  | 19 | Non neonates |
| 14 | 2004^14^ | Switzerland | RCT | 4 | 11 | PAF with CUF |
|  |  |  |  |  | 11 | PAF with MUF |
|  |  |  |  |  | 10 | PSF with CUF |
|  |  |  |  |  | 9 | PSF with MUF |
| 15 | 2005^15^ | Switzerland | Prospective observational cohort | 1 | 40 | Observational study |
| 16 | 2002^16^ | UK | Prospective observational cohort | 2 | 14 | Control |
|  |  |  |  |  | 13 | Steroid treatment |
| 17 | 2002^17^ | Israel | prospective observational cohort | 1 | 10 | Observational study |
| 18 | 2004^18^ | Brazil | Prospective observational cohort | 1 | 14 | Observational study |
| 19 | 2002^19^ | Turkey | RCT | 3 | 10 | Acyanotic |
|  |  |  |  |  | 7 | Cyanotic hyperoxic |
|  |  |  |  |  | 7 | Cyanotic normoxic |
| 20 | 2005^20^ | Switzerland | Prospective observational cohort | 1 | 20 | Observational study |
| 21 | 2017^21^ | Germany | Prospective observational cohort | 1 | 37 | Observational study |
| 22 | 2012^22^ | USA | RCT | 2 | 11 | Placebo |
|  |  |  |  |  | 13 | Ketamine infusion pre CPB |
| 23 | 2007^23^ | Germany | Prospective observational cohort | 1 | 11 | Observational study |
| 24 | 2008^24^ | China | RCT | 2 | 12 | Non pulsatile CPB perfusion |
|  |  |  |  |  | 12 | Pulsatile CPB perfusion |
| 25 | 2006^25^ | China | RCT | 4 | 20 | Conventional MUF |
|  |  |  |  |  | 20 | High flow rate MUF |
|  |  |  |  |  | 20 | High negative pressure MUF |
|  |  |  |  |  | 20 | Long duration high flow rate MUF |
| 26 | 2017^26^ | Saudi Arabia | Prospective observational cohort | 1 | 100 | Observational study |
| 27 | 2013^27^ | Poland | Prospective observational cohort | 1 | 47 | Observational study |
| 28 | 2006^28^ | USA | Prospective observational cohort | 1 | 20 | Observational study |
| 29 | 2016^29^ | Ukraine | Retrospective observational cohort | 1 | 80 | Observational study |
| 30 | 2018^30^ | China | RCT | 3 | 30 | Placebo |
|  |  |  |  |  | 30 | Dexmedetomidine priming |
|  |  |  |  |  | 30 | Dexmedetomidine infusion |
| 31 | 2014^31^ | USA | Prospective observational cohort | 1 | 31 | Observational study |
| 32 | 2017^32^ | China | Prospective observational cohort | 1 | 30 | Observational study |
| 33 | 2009^33^ | USA | Prospective observational cohort | 2 | 21 | No AKI |
|  |  |  |  |  | 18 | AKI |
| 34 | 2015^34^ | USA | Prospective observational cohort | 3 | 50 | No AKI |
|  |  |  |  |  | 32 | AKI stage 1 |
|  |  |  |  |  | 34 | AKI stage 2 or 3 |
| 35 | 2019^35^ | Indonesia | Prospective observational cohort | 2 | 84 | No post-operative infection |
|  |  |  |  |  | 24 | Post-operative infection |
| 36 | 2010^36^ | USA | Prospective observational cohort | 1 | 10 | Observational |
| 37 | 2002^37^ | Turkey | RCT | 2 | 15 | High dose steroid treatment |
|  |  |  |  |  | 15 | Low dose steroid treatment |
| 38 | 2017^38^ | India | RCT | 2 | 50 | St Thomas cardioplegia |
|  |  |  |  |  | 50 | Del Nido cardioplegia |
| 39 | 2004^39^ | Turkey | RCT | 2 | 14 | Conventional ultrafiltration |
|  |  |  |  |  | 13 | MUF |
| 40 | 2010^40^ | Germany | RCT | 2 | 19 | Normal flow CPB |
|  |  |  |  |  | 21 | High flow CPB |
| 41 | 2018^41^ | Germany | Prospective observational cohort | 2 | 31 | No AKI |
|  |  |  |  |  | 28 | AKI |
| 42 | 2016^42^ | Turkey | RCT | 2 | 15 | Placebo |
|  |  |  |  |  | 15 | Steroid treatment |
| 43 | 2020^43^ | USA | RCT | 2 | 12 | Control |
|  |  |  |  |  | 13 | Prophylactic peritoneal drain |
| 44 | 2003^44^ | USA | Prospective observational cohort | 1 | 16 | Observational |
| 45 | 2009^45^ | Sweden | RCT | 2 | 10 | Standard CPB circuit |
|  |  |  |  |  | 10 | Heparin coated CPB circuit |
| 46 | 2014^46^ | India | Prospective observational cohort | 4 | 24 | < 1 year of age |
|  |  |  |  |  | 55 | 1-12 years of age |
|  |  |  |  |  | 30 | 12-18 years of age |
|  |  |  |  |  | NB the fourth cohort was excluded because it comprised of patients > 18 years of age | |
| 47 | 2009^47^ | Germany | Prospective observational cohort | 1 | 20 | Observational |
| 48 | 2010^48^ | USA | Prospective observational cohort | 1 | 10 | Observational |
| 49 | 2007^49^ | USA | Prospective observational cohort | 1 | 20 | Observational |
| 50 | 2014^50^ | USA | Prospective observational cohort | 2 | 22 | Normal serum IgG levels |
|  |  |  |  |  | 25 | Low serum IgG levels |
| 51 | 2010^51^ | USA | Prospective observational cohort | 1 | 9 | Observational study |
| 52 | 2016^52^ | India | RCT | 3 | 13 | Placebo |
|  |  |  |  |  | 13 | Low dose sildenafil preconditioning |
|  |  |  |  |  | 13 | High dose sildenafil preconditioning |
| 53 | 2019^53^ | UK | RCT | 2 | 24 | Control |
|  |  |  |  |  | 15 | RIPC |
| 54 | 2014^54^ | USA | Prospective observational cohort | 1 | 49 | Observational study |
| 55 | 2010^55^ | China | RCT | 2 | 30 | Control |
|  |  |  |  |  | 30 | RIPC |
| 56 | 2006^56^ | Slovenia | Prospective observational cohort | 2 | 5 | Normal CI |
|  |  |  |  |  | 5 | Low CI |
| 57 | 2011^57^ | USA | RCT | 2 | 20 | FX05 Oxygenator |
|  |  |  |  |  | 20 | Terump Capiox RX05 oxygenator |
| 58 | 2014^58^ | Japan | RCT | 2 | 9 | Normoxia |
|  |  |  |  |  | 13 | Hyperoxia |
| 59 | 2017^59^ | Australia | RCT | 3 | 22 | Control |
|  |  |  |  |  | 22 | Ketamine induction |
|  |  |  |  |  | 22 | Ketamine infusion |
| 60 | 2005^60^ | Switzerland | RCT | 2 | 26 | Control |
|  |  |  |  |  | 24 | Steroid treatment |
| 61 | 2011^61^ | USA | RCT | 2 | 9 | No ACEi |
|  |  |  |  |  | 11 | Ongoing ACEi |
| 62 | 2012^62^ | USA | RCT | 2 | 81 | Unwashed blood products |
|  |  |  |  |  | 81 | Washed blood products |
| 63 | 2009^63^ | UK | RCT | 2 | 35 | Normoxia |
|  |  |  |  |  | 32 | Hyperoxia |
| 64 | 2014^64^ | Pakistan | RCT | 2 | 76 | Control |
|  |  |  |  |  | 76 | Steroid treatment |
| 65 | 2015^65^ | China | Retrospective observational cohort | 2 | 47 | ASO |
|  |  |  |  |  | 74 | Norwood procedure |
| 66 | 2012^66^ | USA | Prospective observational cohort | 3 | 29 | CPB and no DSC |
|  |  |  |  |  | 27 | CPB and DSC |
|  |  |  |  |  | NB one cohort was excluded due to the absence of CPB | |
| 67 | 2001^67^ | Denmark | Prospective observational cohort | 2 | 13 | Observational |
| 68 | 2002^68^ | Isreal | Prospective observational cohort | 2 | 16 | Observational |
| 69 | 2020^69^ | Korea | Prospective observational cohort | 2 | 18 | No AKI |
|  |  |  |  |  | 12 | AKI |
| 70 | 2005^70^ | Spain | Prospective observational cohort | 2 | 8 | Neonate |
|  |  |  |  |  | 19 | Non neonate |
| 71 | 2017^71^ | Finland | RCT | 2 | 20 | Placebo |
|  |  |  |  |  | 20 | Steroid treatment |

ASD = atrial septal defect, AI = aortic insufficiency, AS = aortic stenosis, ASO = arterial switch operation, AVSD = atrioventricular septal defect, BCPC = Bidirectional Cavopulmonary Connection, CAT = common arterial trunk, CHD = congenital heart disease, DORV = double outlet right ventricle, HLHS = Hypoplastic Left Heart Syndrome, HTN = hypertension, IAA = interrupted aortic arch, MPA = main pulmonary artery, MS = mitral stenosis, PA = pulmonary atresia, PA IVS = pulmonary atresia intact ventricular septum, RCT = randomized controlled trial, RPA = right pulmonary artery, RV-PA = right ventricle to pulmonary artery, RVOTR = Right ventricular outflow tract reconstruction; SV = single ventricle, TA = Tricuspid Atresia, TAPVC = Total anomalous pulmonary venous connection, TGA = Transposition of the Great Arteries, TOF = Tetralogy of Fallot, VSD = ventricular septal defect, UK = United Kingdome, USA = United States of America,

**Table 2.** Demographic and surgical details of included studies and patients

| ID | Cohorts | Subjects | Cohort Description | Age (months) | Weight (kg) | Males  n (%) | CPB time (minutes) | AXC time (minutes) |
| --- | --- | --- | --- | --- | --- | --- | --- | --- |
| 1 | 2 | 14 | Placebo | 6.7^*^ (IQR 5.6, 8.1) | 7.2^*^ (IQR 5.8, 8.4) | 7 (50) | 66^*^ (IQR 59, 81) | 49^*^(IQR 22, 89) |
|  |  | 15 | Steroid treatment | 4.8^*^ (IQR 8.3) | 7.0^*^ (IQR 6.1, 7.8) | 10 (67) | 55^*^ (IQR 47, 103) | 20^*^ (IQR 7, 64) |
| 2 | 4 | 20 | Control | 25.2^#^ (SD 13.2) | 7.4^#^ (SD 1.6) |  | 62.4^#^ (SD 23.3) | 34.4^#^ (SD 14.4) |
|  |  | 20 | BUF | 29.0^#^ (SD 13.0) | 11.1^#^ (SD 1.7) |  | 58.5^#^ (SD 22.2) | 42.6^#^ (SD 21.7) |
|  |  | 20 | MUF | 26.8^#^ (SD 18.2) | 10.8^#^ (SD 2.2) |  | 67.3^#^ (SD 20.5 | 40.8^#^ (SD 16.1) |
|  |  | 20 | BUF and MUF | 25.8^#^ (SD 15.2) | 9.7^#^ (SD 2.2) |  | 68.9^#^ (SD 22.0) | 42.5^#^ (SD 14.2) |
| 3 | 2 | 30 | Routine therapy | 6.2^#^ (SD 1.6) | 16.8^#^ (SD 8.0) | 18 (60) | 58.0# (SD 2.8) | 32.4^#^ (SD3.8) |
|  |  | 30 | Tight glucose control | 5.7^#^ (SD 2.0) | 18^#^ (SD 4.8) | 16 (53) | 55.0^#^ (SD 3.9) | 31.6^#^ (2.4) |
| 4 | 2 | 13 | Control | 13.4^#^ (SD 7.8) | 7.4^#^ (SD 1.6) | 4 (31) | 118^#^ (SD56) |  |
|  |  | 13 | Sivelestat therapy | 10.8^#^ (SD 5.9) | 7.5^#^ (SD 1.7) | 9 (69) | 118^#^ (SD 58) |  |
| 5 | 2 | 20 | Control |  |  |  |  |  |
|  |  | 20 | Steroid treatment |  |  |  |  |  |
| 6 | 1 | 40 | Observational study | 0.2^*^ (IQR 0.1, 0.3) | 7.5^*^ (range 3.5-24.4) |  | 1333.5^#^ (SD 73.1) |  |
| 7 | 1 | 24 | Observational study | 3.2^*^ (IQR 2.9, 3.6) | 0.23^*^ (IQR 0.17, 0.37) |  | 128^*^ (IQR 119, 138) | 52^*^ (IQR 50, 60) |
| 8 | 2 | 32 | Del Nido cardioplegia | 28.0^#^ (SD 16.4) | 10.3^#^ (SD 3.1) | 17 (53) | 137.5^#^ (SD 36.6) | 96.5^#^ (SD 30.8) |
|  |  | 27 | St Thomas cardioplegia | 24.2^#^ (SD 15.9) | 9.6^#^ (SD 2.8) | 9 (33) | 127.6^#^ (SD 34.2) | 82.0^#^ (SD 23.9) |
| 9 | 2 | 20 | Control | 26.4^#^ (SD 40.8) | 11.5^#^ (SD 10) |  | 88.0^#^ (SD 25) | 59^#^ (SD 13) |
|  |  | 17 | RIPC | 10.8^#^ (SD 10.8) | 9.6^#^ (SD 2.8) |  | 80^#^ (SD 24) | 55^#^ (SD 13) |
| 10 | 2 | 15 | Non coated CPB circuit | 2.9^#^ (SD 2.7) | 4.2^#^ (SD 1.0) | 8 (53) | 108.6^#^ (SD 38*.36)* | 69.7^#^ (SD 26.3) |
|  |  | 13 | Phosphorylcholine-coated CPB circuit | 2.65^#^ (SD 2.82) | 4.2^#^ (SD 1.0) | 3 (23) | 117.5^#^ (SD 48.6) | 68.9^#^ (SD 32.1) |
| 11 | 2 | 84 | Control | 32.3^#^ (SD 15.3) | 10.0^#^ (SD 0.5) | 40 (48) | 108.2^#^ (SD 19.7) | 66.3^#^ (SD 35.7) |
|  |  | 84 | Steroid treatment | 33.2^#^ (SD 18.4) | 10.6^#^ (SD 0.5) | 42 (50) | 105.8^#^ (SD 35.7) | 68.4^#^ (SD 29.0) |
| 12 | 2 | 28 | Normothermia | 90.5^*^ (IQR 42, 127) | BSA only | 9 (32) | 48.5^*^ (IQR 39, 66) | 22^*^ (IQR 14.5, 33.0) |
|  |  | 31 | Hypothermia | 52.0^*^ (IQR 26, 138) | BSA only | 16 (52) | 62.0^*^ (IQR 47, 79) | 32^*^ (IQR 27, 49) |
| 13 | 2 | 8 | Neonates | 0.4^#^ (SD 0.1) | 3.3^#^ (SD 0.4) | 5 (63) | 119.8^#^ (SD 19.7**)** | 63.3^#^ (SD 6.4) |
|  |  | 19 | Non neonates | 51.1^#^ (SD 48.6) | 18.5^#^ (SD 14.6) | 9 (47) | 58.2^#^ (SD 19.2) | 31.1^#^ (SD 12.*) |
| 14 | 4 | 11 | PAF with CUF | 16.8^#^ (SD 16.8) | BSA only |  | 96.8^#^ (SD 30) | 50.7^#^ (SD 22.0) |
|  |  | 11 | PAF with MUF | 30.0^#^ (SD 22.8) | BSA only |  | 97.2^#^ (SD 41) | 54.8^#^ (SD 31) |
|  |  | 10 | PSF with CUF | 15.6^#^ (SD 15.6) | BSA only |  | 106.0^#^ (SD 43) | 56.1^#^ (SD 23) |
|  |  | 9 | PSF with MUF | 19.2^#^ (SD 19.2) | BSA only |  | 95.0^#^ (SD 26) | 42.3^#^ (SD 21) |
| 15 | 1 | 40 | Observational study | 11.0^*^ (range 3.0-83.0) | 7.5^*^ (range 3.4-24.4) |  | 132^*^ (range 67-345) | 39^*^ (range 0-205) |
| 16 | 2 | 14 | Control | 7.5^*^ (IQR 2.5, 9.8) | 6.5^*^ (IQR 4.3, 9.4) |  | 99* (IQ 82, 124) | 65* (IQR 42, 74( |
|  |  | 13 | Steroid treatment | 2^*^ (IQR 0.1, 10.0) | 4.0^*^ (IQR 3.3, 7) |  | 74* (IQR 70, 110) | 48* (IQR 48, 74) |
| 17 | 1 | 10 | Observational study | 52.3^#^ (SD 52.9) | 17.3^#^ (SD 14.3) | 5 (50) | 79.8^#^ (SD 49.9) | 49.2^#^ (SD 36) |
| 18 | 1 | 14 | Observational study | 27^#^ (SD22.1) | 13.2^#^ (SD 3.1) | 7 (50) | 108^#^ (SD 36.7) | 69.6^#^ (SD 29.4) |
| 19 | 3 | 10 | Acyanotic | 21^#^ (SD 17) | 8.8^#^ (SD 2.7) | 5 (50) | 98^#^ (SD 12) | 62^#^ (SD 15) |
|  |  | 7 | Cyanotic hyperoxic | 26^#^ (SD 15) | 9.1^#^ (SD 2.4) | 4 (57) | 115^#^ (SD 24) | 72^#^ (SD 17) |
|  |  | 7 | Cyanotic normoxic | 24^#^ (SD 11) | 11.2# (2.6) | 4 (57) | 118^#^ (SD 22) | 75^#^ (SD 21) |
| 20 | 1 | 20 | Observational study | 27.1^#^ (SD 27.2) | 10.3^#^ (SD 6.5) |  | 119.0^#^ (SD 58.0) | 57.6^#^ (SD 35.9) |
| 21 | 1 | 37 | Observational study | 5.0^*^ (range 0.1-116) |  | 25 (68) |  |  |
| 22 | 2 | 11 | Placebo | 5.4^#^ (SD 2.3) | 5.8^#^ (SD 1.5) | 3 (27) | 70.3^#^ (SD 15.4) | 43.7^#^ (SD 13.1) |
|  |  | 13 | Ketamine infusion pre CPB | 5.4^#^ (SD 2.4) | 5.7^#^ (SD 1.3) | 4 (31) | 74.5^#^ (SD 24.3) | 48.5^#^ (SD 20.3) |
| 23 | 1 | 11 | Observational study | 0.4^*^ (IQR 0.3, 0.4) | 3.2^*^ (IQ 3, 3.3) | 9 (81) | 132^*^ (IQR 123, 144) | 66^*^ (IQR 64, 78) |
| 24 | 2 | 12 | Non pulsatile CPB perfusion | 12.6^#^ (SD 4.3 | 9.5^#^ (SD 2.8) |  | 102^#^ (SD 31.2) | 78.4^#^ (SD 22.2) |
|  |  | 12 | Pulsatile CPB perfusion | 14.3^#^ (SD 5.1) | 9.2^#^ (SD 2.4) |  | 95.6^#^ (SD 24.7) | 72.9^#^ (SD 19.5) |
| 25 | 4 | 20 | Conventional MUF | 6.0^#^ (SD 2.4) | 6.4^#^ (SD 1.1) | 12 (60) | 78.7^#^ (SD26.2) | 49.4^#^ (SD 20.3) |
|  |  | 20 | High flow rate MUF | 6.6^#^ (SD 2.6) | 6.6^#^ (SD 0.8) | 10 (50) | 73.2^#^ (SD21.3) | 43.2^#^ (SD 15.5) |
|  |  | 20 | High negative pressure MUF | 7.1^#^ (SD 2.4) | 6.8^#^ (SD 1.6) | 11 (55) | 68^#^ (SD 19.0) | 42.9^#^ (SD 18.9) |
|  |  | 20 | Long duration high flow rate MUF | 7.2^#^ (SD 3.7) | 6.1^#^ (SD 1.4) | 15 (75) | 75.5^#^ (SD 24.8) | 43.7^#^ (SD 23.2) |
| 26 | 1 | 100 | Observational study | 20.5^#^ (SD 2.7) | 8.7^#^ (SD 0.63) |  | 97.2^#^ (SD 4.4) | 70.7^#^ (SD 3.7) |
| 27 | 1 | 47 | Observational study | 28.6^*^ (range 0.5-204) | 10.9^#^ (range 3.2-53) | 24 (51) | 68^#^ (range 30-131) | 43.2^#^ (range 2-85) |
| 28 | 1 | 20 | Observational study | 15^*^ (range 0.1-180) |  | 11 (55) | 91^*^ (range 42-212) | 54^*^ (range 22-156) |
| 29 | 1 | 80 | Observational study | 0.1^*^ (IQR 0.1-122) | 3.4 (IQR 3.03, 3.8) |  | 150^*^ (IQR 137, 179) | 79^*^ (IQR 71, 87) |
| 30 | 3 | 30 | Placebo | 51.6^*^ (SD 19.2) | 13.3^#^ (SD 3.7) | 17 (56) | 55.4^*^ (SD 11.3) | 31.3^*^ (SD 8.7) |
|  |  | 30 | Dexmedetomidine priming | 44.4^*^ (SD 15.6) | 15.2^#^ (SD 2.8) | 14 (47) | 51.8^*^ (SD 12.2) | 29.1^*^ (SD 7.2) |
|  |  | 30 | Dexmedetomidine infusion | 62.4^*^ (SD 42.0) | 14.2^#^ (SD 3.4) | 17 (56) | 53.3^*^ (SD 12.2) | 30.2^*^ (SD .2) |
| 31 | 1 | 31 | Observational study | 6.0^*^ (IQR 0.1, 122) | 7.0^*^ (IQR 3.1, 54.6) | 14 (45) | 98^*^ (range 28-202) | 66^*^ (IQR 64, 78) |
| 32 | 1 | 30 | Observational study | 12.8^#^ (SD 8.9) | 7.9^#^ (SD 2.4) | 17 (57) | 54.6# (SD 17.6) | 33.6^#^ (SD 15.2) |
| 33 | 2 | 21 | No AKI | 25.1^#^ (SD 24.0) | 87^#^ (SD 44) | 13 (62) | 87^#^ (SD 44) |  |
|  |  | 18 | AKI | 36^#^ (SD 63.4) | 136^#^ (SD 63) | 9 (50) | 136^#^ (SD 63) |  |
| 34 | 3 | 50 | No AKI | 53.0^#^ (SD 64.0) | 19^#^ (SD 22) | 28 (56) | 118^#^ (SD 64) | 57^#^ (SD 51) |
|  |  | 32 | AKI stage 1 | 33^#^ (SD 45) | 12^#^ (SD 12) | 20 (63) | 101^#^ (SD 40) | 46^#^ (SD 41) |
|  |  | 34 | AKI stage 2 or 3 | 13.0^#^ (SD 23.0) | 7^#^ (SD 4.6) | 13 (38) | 134^#^ (SD 80) | 46^#^ (SD 40) |
| 35 | 2 | 84 | No post-operative infection | 7.2^*^ (Range 1.8-12.1) | 5.3^*^ (range 2.1-8.9) | 40 (48) | 69.0^*^ (IQR 33, 183) | 32^#^ (range 3-142) |
|  |  | 24 | Post-operative infection | 3.6^*^ (range 0.1-12) | 4.5^*^ (range 2.3-7.1) | 14 (58) | 148.5^*^ (range 37-265) | 87.0^#^ (range 5-170) |
| 36 | 1 | 10 | Observational | 15.4^#^ (SD 14.1) | 8.0^#^ (SD 3.5) |  | 126.7^#^ (SD 4.7) | 69.9^#^ (SD 44.1) |
| 37 | 2 | 15 | High dose steroid treatment | 40.8^#^ (SD 31.2) | 12.4^#^ (SD 6.7) |  | 64.7^#^ (SD 15.9) | 45.7^#^ (SD 12.8) |
|  |  | 15 | Low dose steroid treatment | 54^#^ (SD 45.6) | 15^#^ (SD 8.9) |  | 711.6^#^ (SD 33.9) | 48.7^#^ (SD 12.8) |
| 38 | 2 | 50 | St Thomas cardioplegia | 64.8^#^ (SD 37.3) | 15.7^#^ (SD 10.0) | 33 (66) | 77.7^#^ (SD 20.2) | 48.7^#^ (#16.3) |
|  |  | 50 | Del Nido cardioplegia | 51.6^#^ (SD 35.8) | 14.7^#^ (SD 9.1) | 38 (76) | 66.6^#^ (SD 15.5) | 41.1^#^ (SD 13.3) |
| 39 | 2 | 14 | Conventional ultrafiltration | 13.0^#^ (SD 13.0) | 7.3^#^ (SD 0.1) | 10 (71) | 111.8^#^ (SD 19.6) | 78.2^#^ (SD 21.4) |
|  |  | 13 | MUF | 9.4^#^ (SD 2.0) | 7.3^#^ (SD 0.7) | 8 (62) | 102.8^#^ (SD 19.3) | 7**2.8**^#^ **(SD 18.5)** |
| 40 | 2 | 19 | Normal flow CPB | 8.1^#^ (SD 8.2) | 6.7^#^ (SD 3.3) |  | 104.7^#^ (SD 47.8) | 63.2^#^ (SD 35) |
|  |  | 21 | High flow CPB | 16.8^#^ (SD 21) | 8.3^#^ (SD 5) |  | 100.2^#^ (SD 39) | 53.5^#^ (SD 32) |
| 41 | 2 | 31 | No AKI | 3.5^*^ (range 0.3, 10.2) | 4.5^*^ (Range 2.307.5) |  | 77^*^ (range 23-160) | 47^*^ (range 0-110) |
|  |  | 28 | AKI | 0.9^*^ (range 0.2-9.1) | 3.4 (range 2.5-9.1) |  | 75^*^ (range 30-150) | 75^*^ (range 30-150) |
| 42 | 2 | 15 | Placebo | 26.3^#^ (SD 15.6) | 13.8^#^ (SD 5.6) | 9 (60) | 75.4^#^ (SD 33.7) | 55.4^#^ (SD 27.8) |
|  |  | 15 | Steroid treatment | 27.6^#^ (SD 18) | 14.1^#^ (SD 8.3) | 8 (53) | 90.2^#^ (SD 34) | 66.3^#^ (SD 40.9) |
| 43 | 2 | 12 | Control | 0.2* (IQR 0.1, 0.5) | 3.3^*^ (IQR 3.0, 3.6) | 8 (67) | 218.5 (IQR 182.5, 292.5) | 111^*^ (IQR 74.7, 134.3) |
|  |  | 13 | Prophylactic peritoneal drain | 0.23^*^ (IQR 0.2, 0.4) | 3.0^*^ (IQR 2.6, 3.5) | 10 (77) | 271^*^ (IQR 234 310) | 122.5^*^ (IQR 94.3, 196.0) |
| 44 | 1 | 16 | Observational | 0.9^#^ (SD 0.23) |  |  | 140^#^ (SD 15) | 88.0^#^ (SD 11) |
| 45 | 2 | 10 | Standard CPB circuit | 11^*^ (range 5-16.5) | 7.6^*^ (range 3.8-12) |  | 106^*^ (IQR 90, 146) |  |
|  |  | 10 | Heparin coated CPB circuit | 7^*^ (range 3.5-21) | 7.0* (range 3.2-12.5) |  | 125^*^ (IQR 96, 150) |  |
| 46 | 3 | 24 | < 1 year of age | 8.3^#^ (SD 2.6) | BMI only | 13 (54) | 90.6^#^ (SD 8.7) |  |
|  |  | 55 | 1-12 years of age | 63.6^#^ (SD 39.6) | BMI only | 30 (55) | 90.5^#^ (SD 0.58) |  |
|  |  | 30 | 12-18 years of age | 189.6^#^ (SD 19.2) | BMI only | 18 60) | 86.1^*^ (SD 8.5) |  |
|  |  | *NB the fourth cohort was excluded because it comprised of patients > 18 years of age* | | | | | | |
| 47 | 1 | 20 | Observational | 5.5^#^ (SD 6.4) | 6.5^#^ (SD 3.2) | 13 (65) | 140.0^#^ (SD 59.0) | 57.0^#^ (SD 27) |
| 48 | 1 | 10 | Observational | 17.4^#^ (SD 16.9) | 8.0^#^ (SD 3.5) | 1 (10) | 126.7^#^ (SD 45.6) | 69.9^#^ (SD 44.1) |
| 49 | 1 | 20 | Observational | 9.0^#^ (SD 11.3) | 6.3^#^ (SD 4.1) | 10 (50) | 94.0^#^ (SD 35.0) | 59.0^#^ (SD 29) |
| 50 | 2 | 22 | Normal serum IgG levels | 0.23^#^ (IQR 0.097, 0.12) | 3.2^#^ (IQR 2.9, 3.6) | 32 (68) | 168^#^ (SD 58) |  |
|  |  | 25 | Low serum IgG levels |  |  |  | 180.0^#^ (SD 34.0) |  |
| 51 | 1 | 9 | Observational study | 4.3^#^ (SD 0.5) |  | 4 (44) | 95.7^#^ (SD 7.4) | 57.2^#^ (SD 5) |
| 52 | 3 | 13 | Placebo | 36^*^ (range 12-192) | 14^*^ (IQR 0, 31) |  | 57^*^ (IQR 37, 77) | 27^*^ (IQR 14, 40) |
|  |  | 13 | Low dose sildenafil preconditioning | 48^*^ (range 12-168) | 12^*^ (IQR 4, 20) |  | 51^*^ (IQR 41, 61) | 27^*^ (IQR 14, 40) |
|  |  | 13 | High dose sildenafil preconditioning | 36^*^ (IQR 0, 96) | 11^*^ (IQR 1, 21) |  | 51^*^ (IQR 41, 61) | 27^*^ (IQR 22, 32) |
| 53 | 2 | 24 | Control | 9^*^ (IQR 5, 56) | 8.3^*^ (IQR 6.3, 17.8) | 11 (46) | 151^*^ (IQR 191, 213) | 86^*^ (IQR 23, 131) |
|  |  | 15 | RIPC | 19^*^ (IQR 6, 58) | 12.2^*^ (IQR 6.8, 17.4) | 13 (87) | 142^*^ (IQ 104, 202) | 69^*^ (IQR 41, 133) |
| 54 | 1 | 49 | Observational study | 5.1* (IQR 1.7, 34.2) | 6.0^*^ (IQR 4.9, 12.2) | 23 (47) | 114^*^ (IQR 76, 150.5) | 69.6^*^ (IQR43.5, 92) |
| 55 | 2 | 30 | Control | 5.1^#^ (SD 1.9) | 5.6^#^ (SD 0.92) | 16 (53) | 33.8^#^ (SD 9.6) | 24.1^#^ (SD 8.2) |
|  |  | 30 | RIPC | 5.4^#^ (SD 2.0) | 5.8^#^ (SD 1.0) | 18 (60) | 34.5^#^ (SD 10.9) | 24.1^#^ (SD 9.8) |
| 56 | 2 | 5 | Normal CI | 18.4^*^ (IQR 16.8, 20.2) | 10.0^*^ (IQR 9.5, 11.0) | 3 (60) | 83^*^ (IQR 70, 102) |  |
|  |  | 5 | Low CI | 19.0^*^ (IQR 16.5, 24.1) | 8.6^*^ (IQR 1.7, 1.1) | 2 (40) | 76^*^ (IQR 71, 119) |  |
| 57 | 2 | 20 | FX05 Oxygenator |  | 5.3^#^ (SD 1.7) |  | 94^#^ (SD 39) | 42^#^ (SD 30) |
|  |  | 20 | Terump Capiox RX05 oxygenator |  | 5.5^#^ (SD 1.5) |  | 82^#^ (SD 32) | 32.0^#^ (SD 29) |
| 58 | 2 | 9 | Normoxia | 23.2^#^ (SD 27.7) | 8.9^#^ (SD 5.5) |  | 126^#^ (SD 47.2) | 78.9^#^ (SD 36.4) |
|  |  | 13 | Hyperoxia | 11.6^#^ (SD 18.8) | 6.9^#^ (SD 4.5) |  | 118^#^ (SD 28.2) | 68.9^#^ (SD 23.9) |
| 59 | 3 | 22 | Control | 34.7^#^ (SD 10.2) | 11.5^#^ (SD 2.4) | 11 (50) | 98.7^#^ (SD47.5) |  |
|  |  | 22 | Ketamine induction | 32.0^#^ (SD 7.3) | 10.5^#^ SD 1.5) | 14 (64) | 69.8^#^ (SD28.6) |  |
|  |  | 22 | Ketamine infusion | 22.9^*^ (SD 5.0) | 9.8^#^ (SD 1.3) | 10 (45) | 73.5^#^ (SD 29.8) |  |
| 60 | 2 | 26 | Control | 21.5^*^ (IQR 8.3, 48.8) | 11.4^*^ (IQR 7.5, 15.5) |  | 110^*^ (IQR 85, 136) | 42^*^ (IQR 18, 53) |
|  |  | 24 | Steroid treatment | 5^*^ (IQR 4, 7.3) | 5.8^*^ (IQR 4.6, 6.4) |  | 134^#^ (IQR 103, 182) | 44^*^ (IQR 0, 61) |
| 61 | 2 | 9 | No ACEi | 20.8^#^ (SD 6.5) | 9.1^#^ (SD 1.2) | 6 (67) | 113.7^#^ (SD 23.9) | 23.7^#^ (SD 12.2) |
|  |  | 11 | Ongoing ACEi | 13.7^#^ (SD 3.5) | 7.8^#^ (SD 1.2) | 7 (63) | 138.4^#^ (SD 19.5) | 64.6^#^ (SD 21.8) |
| 62 | 2 | 81 | Unwashed blood products | 7^*^ (range 0.1-204) | 6.7^*^ (range 2.2-66) | 64 (79) | 98^*^ (Range 28-290) | 61^*^ (Range 2-216) |
|  |  | 81 | Washed blood products | 6^*^ (range 0.1-510) | 6.8^*^ (range 2.8-106) | 63 (78) | 95^*^ (range 25-356) | 62^*^ (Range 6-234) |
| 63 | 2 | 35 | Normoxia | 15^*^ (IQR 6, 47) | 9.6^*^ (IQR 7.5, 13.9) | 22 (63) | 82.0^*^ (Range 41-147) | 75^#^ (SD 26) |
|  |  | 32 | Hyperoxia | 14.5^*^ (IQR 5, 51) | 9.5^*^ (IQR 6.6, 15.8) | 18 (56) | 89.7^*^ (range 43-210) | 52.3^#^ (SD 24.7) |
| 64 | 2 | 76 | Control | 48^*^ (IQR 78) |  | 43 (57) | 100^*^ (IQ 25, 175) | 65^*^ (range 1-129) |
|  |  | 76 | Steroid treatment | 31^*^ (IQR 62) |  | 41 (54) | 100^*^ (IQR 33, 177) | 63^*^ (IQR 0, 129) |
| 65 | 2 | 47 | ASO | 0.3^#^ (SD 0.2) | 3.6^#^ (SD 0.5) |  | 142^#^ (SD 76) | 75^#^ (SD 26) |
|  |  | 74 | Norwood procedure | 0.5^#^ (SD 0.4) | 3.3^#^ (SD 0.5) |  | 107^#^ (SD 36) | 43^#^ (SD 19) |
| 66 | 3 | 29 | CPB and no DSC | 1.6^*^ (IQR 0.3, 2.2) | 3.4^*^ (IQR 3.1, 4) | 16 (55) | 130^*^ (IQR 94, 176) | 86^*^ (IQR 61, 113) |
|  |  | 27 | CPB and DSC | 0.2^*^ (IQR 0.1, 0.6) | 3.1^*^ (IQR 2.8, 3.4) | 18 (67) | 167^*^ (IQR 153, 198) | 76^*^ (IQR 56, 93) |
|  |  | *NB one cohort was excluded due to the absence of CPB* | | | | | | |
| 67 | 2 | 13 | Observational | 6.2^#^ (SD 3.2) | 6.7^*^(IQR 2.8, 10.6) |  | 93.8^#^ (SD 35.5) | 53^*^ (IQR 15, 91) |
| 68 | 2 | 16 | Observational | 75.6^#^ (SD 60) |  | 10 (63) | 93^*^ (range 25-299) | 64^*^ (range 9-202 |
| 69 | 2 | 18 | No AKI | 1.14^*^ (IQR 0.6, 3.61) |  | 11 (61) | 58^*^ (IQR 48.5, 91.5) | 39^*^ (IQR 30, 59.5) |
|  |  | 12 | AKI |  |  | 9 (75) | 83.5^*^ (IQR 76, 136) | 57^*^ (IQR 44, 79) |
| 70 | 2 | 8 | Neonate | 0.4^#^ (SD 0.1) | 3.3^#^ (SD 0.4) | 5 (63) | 119.8^#^ (SD 19.7) | 63.3^#^ (SD 6.4) |
|  |  | 19 | Non neonate | 51.1^#^ (SD 48.4) | 18.5^#^ (SD 14.6) | 9 (47) | 58.2^#^ (SD 19.2) | 31.1^#^ (12.8) |
| 71 | 2 | 20 | Placebo | 0.3^#^ (SD 0.2) | 3.5^#^ (SD (0.6) | 14 (70) | 161.5^#^ (SD 61.5) | 88.3^#^ (SD 42.5) |
|  |  | 20 | Steroid treatment | 0.3^#^ (SD 0.1) | 3.4^#^ (SD 0.5) | 15 (75) | 180.6^#^ (SD 47) | 103.4^#^ (SD 35.3) |

Median^*^; mean^#^

*ACEi = angiotensin AKI = acute kidney injury; ASO = arterial switch operation; AXC = aorta cross clamp; BUF = balanced ultrafiltration; CI = cardiac index; CPB = cardiopulmonary bypass; IQR = interquartile range, MUF = modified ultrafiltration; PAF = polyamid*

*Filter; PSF = polyarylethersulfon filters; RIPC = remote ischaemic perfusion conditioning; SD = standard deviation*

**Table 3.** Outcome data, where reported represented in individual studies

| Study | Cohort | ICU LOS | | Mechanical ventilation | | Hospital LOS | |
| --- | --- | --- | --- | --- | --- | --- | --- |
|  |  | Days | P value | Hours | P value | Days | P value |
| 4 | Placebo | 2* (IQR 2, 5) | 0.96 |  |  |  |  |
|  | Steroids | 2* (IQR 2, 4) |  |  |  |  |  |
| 8 | Routine | 2.72# (SD 0.50) | < 0.05 | 7.2# (SD 1.6) | <0.05 |  |  |
|  | Insulin | 2.04# (SD 0.54) |  | 10.3# (SD 1.8) |  |  |  |
| 9 | Control | 13.8* (SD 10.6) | 0.39 | 29.8# (SD 106) | 0.7 |  |  |
|  | Sivelestat | 9.0* (SD 16.7) |  | 46.5# (SD 114) |  |  |  |
| 11 | Non cyanotic lesion |  |  | 59.9# (SD 43.4) | 0.0009 |  |  |
|  | Cyanotic lesion |  |  | 179.6# (SD 151.5) |  |  |  |
| 10 | Control | 9* (IQR 5, 11) | 0.31 | 144* (IQR 72, 240) | 0.28 |  |  |
|  | Steroids | 7* (IQR 5, 8) |  | 120* (IQR 196, 144) |  |  |  |
| 12 | TGA | 4* (IQR 3, 6) | 0.31 | 46* (IQR 32, 68) | 0.12 |  |  |
|  | HLHS | 6* (IQR 5, 8) |  | 71* (IQR 46, 90) |  |  |  |
| 13 | Del Nido | 3.51# (SD 4.83) | 0.78 | 37.8# (SD 122.3) | 0.51 |  |  |
|  | St Thomas | 3.21# (SD 2.79) |  | 20.8# (SD 22.4) |  |  |  |
| 15 | Control | 3.6# (SD 2.1) | 0.5 | 56# (SD 3) | 0.6 |  |  |
|  | Coated CPB | 4.9# (SD 3.6) |  | 78# (SD 56) |  |  |  |
| 18 | Normothermia |  |  | 8.5* (IQR 6, 21) |  | 5# (IQR 5, 6) |  |
|  | Hypothermia |  |  | 8* (IQR 6, 23) |  | 5# (IQR 5, 7) |  |
| 20 | Neonate | 10.63# (SD 3.29) | 0.007 | 150# (SD 43.9) | 0.31 |  |  |
|  | Non-neonate | 5.84# (SD 4.07) |  | 53.8# (SD 74.4) |  |  |  |
| 21 | PAF with CUF | 4.8# (SD 2.1) | <0.05 | 43.2# (SD 2.1) |  |  | <0.05 |
|  | PAF with MUF |  |  | 55.6# (SD 557.5) |  |  |  |
|  | PSF with CUF |  |  | 61.9# (SD 46) |  | 21.8# (SD 19.9) |  |
|  | PSF with MUF | 9.0# (SD 7.4) |  | 80.5# (SD 110.2) |  | 11.5# (SD 3.0) |  |
| 25 | Entire cohort | 6# (SD 2.6) |  | 60# (36.24) |  |  |  |
| 26 | Entire cohort | 3.6# (SD 0.76) |  | 23.3# (SD 10.6) |  |  |  |
| 27 | Acyanotic | 3.54# (SD 2.69) |  | 28.6# (SD 28.9) |  |  |  |
|  | Cyanotic hyperoxia | 4.79# (SD 4.6) |  | 42.3# (SD 40.1) |  |  |  |
|  | Cyanotic normoxia | 5.08# (SD 4.92) |  | 45.6# (SD43.5) |  |  |  |
| 28 | Entire Cohort |  |  | 95.1# (SD 120.3) |  |  |  |
| 30 | Control | 3.28# (SD 1.33) | 0.57 | 25.1# (SD 36.8) | 0.34 |  |  |
|  | Ketamine | 3.01# (SD 1.01) |  | 14.0# (Sd 14.99) |  |  |  |
| 36 | Entire cohort | 5.0* (IQR, 2, 90) |  | 48* (IQ 0, 90) |  | 8# (Range 3-90) |  |
| 37 | Entire cohort | 6* (IQR 5, 8) |  | 38* (IQR 34, 68) |  | 16* (IQR 14, 22) |  |
| 39 | Entire cohort | 3.92* (1.13, 35.83) |  |  |  |  |  |
| 40 | Entire cohort | 4.1# (SD 1.5) |  | 12.6# (SD 7.9) |  | 17.8# (SD 5.8) |  |
| 41 | No AKI |  |  |  |  | 4* (IQR 3, 6) |  |
|  | AKI |  |  |  |  | 10.5* (IQR 8, 30) |  |
| 43 | No AKI | 3.0* (IQR 2, 4) | 0.003 |  |  | 6* (IQR 5, 10) | 0.002 |
|  | AKI stage 1 | 3.0* (IQR 2, 5.5) |  |  |  | 5.5* (IQR 4, 12.5) |  |
|  | AKI stage 2 or 3 | 5.5* (IQR 3, 9) |  |  |  | 12* (IQR 9, 15) |  |
| 46 | High dose steroid | 2.75# (SD 2.0) | 0.71 | 9# (SD 5.2) | 0.69 |  |  |
|  | Low dose steroid | 2.59# (SD 1.65) |  | 10# (SD 11.5) |  |  |  |
| 47 | St Thomas cardioplegia | 2.39# (SD 1.8) | 0.03 | 12.1# (SD 15.3) | 0.03 | 6.8# (SD 1.38) | < 0.001 |
|  | Del Nido cardioplegia | 1.69# (SD 0.61) |  | 7# (SD 4.2) |  | 6.0* (SD 0.9) |  |
| 48 | CUF | 3.42* (SD 2.56) | 0.014 | 50.6# (SD 52.0) | 0.017 |  |  |
|  | MUF | 1.62* (SD 094) |  | 19.8# (SD 19.8) |  |  |  |
| 52 | Placebo | 4.0* (SD 1.0) | 0.384 | 10.6# (SD 3.4) | 0.001 | 7# (SD 2) | 0.384 |
|  | Steroid treatment | 6.0* (SD 2.0) |  | 6.1# (SD 2.4) |  | 6# (SD 2) |  |
| 53 | Control |  |  | 91.0* (IQR 64.1, 163.0) | 0.19 | 37.5* (IQR 16.3, 78.8) | 0.69 |
|  | Prophylactic peritoneal drain |  |  | 160.1* (IQR 103.4, 367.0) |  | 33* (IQR 17, 58.5) |  |
| 55 | Standard CPB circuit | 1.5* (IQR 1.0, 8.0) | 0.20 | 24* (IQR 8, 73) | 0.29 |  |  |
|  | Heparin coated CPB circuit | 4.0* (IQR 1.0, 11.0) |  | 57* (IQR 19, 93) |  |  |  |
| 56 | < 1 year of age | 3.5# (SD 1.2) | 0.007 | 12.8# (SD 3.4) | 0.001 | 10.4# (SD 2.8) | 0.026 |
|  | 1-12 years of age | 4.1# (SD 1.3) |  | 11.7# (SD 4) |  | 8.7# (SD 2.8) |  |
|  | 12-18 years of age | 3.4# (SD 1.6) |  | 11.2# (SD 3.2) |  | 8.7# (SD 2.7) |  |
|  | Cohort 4 excluded because > 18 years of age | |  |  |  |  |  |
| 59 | Entire cohort | 5.1# (SD 3.0) |  | 31.2# (SD 21.6) |  |  |  |
| 61 | Normal serum IgG levels | 7.0# (SD 3.0) | 0.001 | 69* (IQR 38, 100) | 0.013 |  |  |
|  | Low serum IgG levels | 16.0# (SD 3.0) |  | 158* (IQR 91, 296) |  |  |  |
| 64 | Placebo | 1.29* (IQR 0.67, 9.0) | 0.386 | 3.62* (range 1.3-167.5) | 0.099 |  |  |
|  | Low dose sildenafil preconditioning | 1.21* (IQR 1.0, 2.33) |  | 1.52* (range 1.0-35.8) |  |  |  |
|  | High dose sildenafil preconditioning | 1.71* (IQR 1.0, 8.04) |  | 0.68* (range 0.4-3.2) |  |  |  |
| 65 | Control | 4.93* (IQR 1.62, 7.2) | 0.029 | 67.0* (IQR 11.0, 115.9) | 0.15 |  |  |
|  | RIPC | 1.79* (IQR 0.93, 3.88) |  | 8.64* (IQR, 2.4, 82.6) |  |  |  |
| 66 | Entire cohort | 4.0* (IQ 2.0, 35.0) |  |  |  |  |  |
| 67 | Control | 5.47# (SD 1.53) |  | 61.1# (SD 30.14) |  |  |  |
|  | RIPC | 5.1# (SD2.43) |  | 66.2# (SD 51.9) |  |  |  |
| 68 | Normal CI | 3.0* (IQR 2.13, 3.75) |  | 22* (IQR 20, 28) |  |  |  |
|  | Low CI | 3.75* (IQR 2.91, 3.92) |  | 24* (IQR 21, 69) |  |  |  |
| 70 | FX05 Oxygenator | 2.08* (IQR 1.83, 10.21) | 0.72 | 21* (IQR 11, 125) | 0.96 | 6.96* (IQR 5.1, 14.8) | 0.25 |
|  | Terump Capiox RX05 oxygenator | 3.04* (IQ 1.38, 6.29) |  | 23* (IQR 9, 31) |  | 5.4* (IQR 4.8, 9.7) |  |
| 72 | Normoxia |  |  | 10.7* (SD 13.4) | 0.22 |  |  |
|  | Hyperoxia |  |  | 27.8* (SD 36.5) |  |  |  |
| 75 | Entire cohort |  |  | 58* (range 29.3-93.8) | 0.34 |  |  |
| 76 | No ACEi |  |  | 36.4# (SD 18.7**)** | 0.32 |  |  |
|  | ACEi |  |  | 89.2# (SD 30.1) |  |  |  |
| 77 | Unwashed blood products | 8.9# (SD 12.36) | 0.86 | 51.5* (range 3-1200) | 0.628 | 8.5* (range 4.0-56.0) | 0.25 |
|  | Washed blood products | 8.6# (SD 10.8) |  | 45.0* (range 4-1008) |  | 5.4* (range 4.8-9.7) |  |
| 78 | Normoxic |  |  | 0.36* (range 0.033-3.18) | 0.66 | 10.5* (IQR 6.0, 14.0) | 0.65 |
|  | Hyperoxic |  |  | 0.33* (0.05-1.2) |  | 8.5* (IQR 6.5, 12.5) |  |
| 81 | ASO | 6.0# (SD 5.0) | NS |  |  | 19# (SD 8) | <0.001 |
|  | Norwood procedure | 7.0# (SD 4.0) |  |  |  | 45# (SD 24) |  |
| 82 | CPB and no DSC | 4.0* (IQR 3.0, 7.0) | <0.0001 | 40* (IQR 18, 72) | < 0.01 | 10* (7, 14) | < 0.0005 |
|  | CPB and DSC | 9.0* (IQR 6, 15) |  | 117* (IQR 74, 165) |  | 21* (IQR 13, 28) |  |
|  | NB cohort 3 was excluded due to the absence of CPB | |  |  |  |  |  |
| 84 | Entire cohort |  |  | 57.6# (SD 31.2) |  |  |  |
| 86 | Neonate | 10.63# (SD 3.29) | 0.007 | 514.6# (SD 202.3) | 0.002 |  |  |
|  | Non neonate | 5.84# (SD 4.07) |  | 53.76# (SD 74.4) |  |  |  |

**Table 4.** A tabular description of the number of studies that examine the specific cytokine based on the time interval

|  | Pre CPB | | On CPB | | | | Post CPB | | | | | | | | | |
| --- | --- | --- | --- | --- | --- | --- | --- | --- | --- | --- | --- | --- | --- | --- | --- | --- |
|  | Baseline | Induction | Start | Mid | End | MUF | 0-2hr | 3-4hr | 6-8hr | 8-12hr | 13-20hr | 1 day | 2 days | 3 days | 4 days | 5 days |
| CRP | 5 | 14 | 3 | 4 | 11 | 1 | 9 | 7 | 8 | 5 | 1 | 13 | 9 | 7 | 4 | 2 |
| IL-6 | 11 | 32 | 8 | 6 | 25 | 5 | 19 | 10 | 15 | 11 | 4 | 33 | 11 | 11 | 4 | 2 |
| IL-7 |  | 1 | 1 |  | 1 |  | 1 |  |  |  |  | 1 |  |  |  |  |
| IL-8 | 8 | 20 | 6 | 7 | 16 | 1 | 14 | 7 | 7 | 8 | 1 | 23 | 9 | 6 | 1 | 1 |
| IL-10 | 8 | 19 | 4 | 5 | 17 | 2 | 10 | 8 | 9 | 6 | 2 | 18 | 4 | 6 | 2 | 1 |
| TNFa | 6 | 18 | 4 | 7 | 12 | 5 | 9 | 4 | 6 | 5 | 3 | 16 | 6 | 5 | 1 | 0 |
| IL-1 | 2 | 1 |  |  |  |  |  |  |  |  |  | 1 | 1 | 1 |  |  |
| IL1-b |  | 5 | 2 | 1 | 4 |  | 2 | 1 |  | 1 |  | 4 | 3 | 1 |  |  |
| IL-2 | 2 | 4 | 2 | 2 | 3 |  | 2 |  | 1 | 1 |  | 3 |  |  |  |  |
| IL-12(p70) |  | 2 | 1 |  | 2 |  | 1 | 1 |  |  |  | 2 | 1 |  |  |  |
| IL-12(p40) |  | 4 | 2 | 1 | 3 |  | 2 |  |  | 1 |  | 3 | 2 | 1 |  |  |
| IL-13 |  | 2 | 1 |  | 1 |  | 1 |  |  |  |  | 2 |  |  |  |  |
| IL-15 |  | 2 | 1 |  | 1 |  | 1 |  |  |  |  | 2 |  |  |  |  |
| IL-16 |  | 2 | 1 |  | 1 |  | 1 |  |  |  |  | 2 |  |  |  |  |
| IL-18 | 1 | 2 | 1 |  | 2 |  | 1 |  | 2 |  |  | 1 |  |  |  |  |
| MCP-1a |  | 4 | 2 | 1 | 1 |  | 3 | 2 |  |  |  | 2 |  |  |  |  |
| ENA-78 |  | 1 |  | 1 |  |  | 1 | 1 |  |  |  |  |  |  |  |  |
| INFy | 1 | 4 | 2 | 1 | 2 |  | 3 |  |  | 1 |  | 3 |  | 1 |  |  |
| EGF |  | 1 | 1 |  | 1 |  | 1 |  |  |  |  | 1 |  |  |  |  |
| sCD40L |  | 1 | 1 |  | 1 |  | 1 |  |  |  |  | 1 |  |  |  |  |
| Eotaxin |  | 2 | 1 |  | 1 |  | 1 |  |  |  |  | 2 |  |  |  |  |
| FGF |  | 1 | 1 |  | 1 |  | 1 |  |  |  |  | 1 |  |  |  |  |
| G-CSF |  | 2 | 1 |  | 1 |  | 1 |  |  |  |  | 2 |  |  |  |  |
| M-CSF | 1 |  |  |  |  |  |  |  |  |  |  |  |  |  |  |  |
| MDC (ccl22) |  | 2 | 1 |  | 1 |  | 1 |  |  |  |  | 2 |  |  |  |  |
| MIP-1a |  | 1 | 1 |  | 1 |  | 1 |  |  |  |  | 1 |  |  |  |  |
| MIP-1b |  | 2 | 1 |  | 1 |  | 1 |  |  |  |  | 2 |  |  |  |  |
| INF-a |  | 1 | 1 |  | 1 |  | 1 |  |  |  |  | 1 |  |  |  |  |
| TNFb |  | 2 | 2 | 1 | 2 |  | 2 |  |  | 1 |  | 1 |  |  |  |  |
| VEGF-A |  | 2 | 1 |  | 1 |  | 1 |  |  |  |  | 2 |  |  |  |  |
| ENA-78 |  | 1 | 1 |  | 1 |  | 1 |  |  |  |  | 1 |  |  |  |  |
| SCF |  | 2 | 1 |  | 1 |  | 1 |  |  |  |  | 2 |  |  |  |  |

NB not all of the presented time points had data that could be included in the meta-analysis (ie some were only available in graphic form)

**Table 5.** Summary of risk-of-bias assessments by study design

| **Assessment tool** | **Study type** | **n** | **Overall judgment** | **Recurrent concerns** |
| --- | --- | --- | --- | --- |
| RoB 2.0 | Randomized interventional studies | 34 | Mostly low/some concerns | Allocation concealment or blinding incompletely reported; selective cytokine reporting |
| ROBINS-I | Non-randomized interventional/comparative studies | 4 | Moderate/serious concerns | Confounding, intervention classification, incomplete adjustment |
| QUIPS | Prospective observational/descriptive studies | 31 | Low/moderate concerns | Confounding, heterogeneous case mix, incomplete adjustment |
| Adapted observational ROBINS-I | Retrospective observational studies | 2 | Moderate/serious concerns | Retrospective design, missing data, confounding |

**Legend:** Design-specific risk-of-bias tools were used: RoB 2.0 for randomized interventional studies, ROBINS-I for non-randomized interventional or comparative studies, and QUIPS for prospective observational or prognostic/descriptive studies. Retrospective observational studies were assessed using an adapted observational approach. Recurrent concerns included incomplete reporting of allocation concealment or blinding, selective biomarker reporting, confounding, heterogeneous case mix, and limited adjustment for perioperative factors. Full study-level assessments are available on request.

**Table 6.** GRADE summary of certainty in the evidence for interleukin-6 (IL-6) trajectories following pediatric cardiopulmonary bypass.

| **Time Point** | **Pooled Estimate (pg/ml)** | **Lower CI** | **Upper CI** | **Number of Cohorts** | **RCTs** | **Observational Studies** | **GRADE Rating** | **Rationale** |
| --- | --- | --- | --- | --- | --- | --- | --- | --- |
| Baseline | 22.77 | 7.76 | 66.75 | 63 | 18 | 13 | Moderate | Multiple contributing cohorts, including RCT-derived cohorts; downgraded for baseline heterogeneity and wide confidence interval. |
| Post CPB | 233.02 | 40.49 | 1340.98 | 35 | 12 | 5 | Low | Predominantly RCT-derived cohorts, but certainty limited by wide confidence interval, substantial heterogeneity, and limited generalizability of the absolute pooled concentration. |
| 0-2 hours | 60.32 | 21.78 | 167.1 | 15 | 3 | 4 | Low | Mixed RCT and observational evidence with plausible early postoperative elevation; downgraded for wide confidence interval, heterogeneity, and fewer RCT-derived cohorts. |
| 3-4 hours | 182.65 | 100.94 | 330.5 | 12 | 4 | 3 | Moderate | Methodologically sound RCT-derived cohorts support early postoperative IL-6 elevation; downgraded for heterogeneity and assay/sampling variability. |
| 6-8 hours | 132.42 | 82.92 | 211.46 | 25 | 9 | 3 | Moderate | Multiple RCT-derived cohorts support sustained IL-6 elevation; downgraded for substantial expected clinical and methodological heterogeneity. |
| 12 hours | 121.66 | 73.44 | 201.52 | 8 | 3 | 2 | Moderate | RCT-derived evidence supports persistent postoperative elevation, but certainty limited by fewer cohorts and heterogeneity. |
| 18-20 hours | 96.46 | 75.7 | 122.91 | 5 | 2 | 0 | Moderate | Few contributing cohorts; RCT-derived data but limited generalizability. |
| POD 1 | 71.29 | 44.55 | 114.09 | 33 | 11 | 7 | Moderate | Multiple cohorts, many RCT-derived, support declining but persistent postoperative elevation; downgraded for heterogeneity and assay/sampling variability. |
| POD 2 | 61.55 | 37.94 | 99.85 | 6 | 1 | 2 | Low | Few cohorts and predominantly observational evidence; limited precision and generalizability. |

**Table 7.** GRADE summary of certainty in the evidence for C-reactive protein (CRP) trajectories following pediatric cardiopulmonary bypass.

| **Time Point** | **Pooled Estimate (pg/ml)** | **Lower CI** | **Upper CI** | **Number of Cohorts** | **RCTs** | **Observational Studies** | **GRADE Rating** | **Rationale** |
| --- | --- | --- | --- | --- | --- | --- | --- | --- |
| Baseline | 0.67 | 0.31 | 1.45 | 7 | 2 | 5 | Low | Mixed study designs with baseline heterogeneity and limited precision |
| Post CPB | 0.09 | 0.05 | 0.15 | 1 | 1 | 0 | Low | Single RCT-derived estimate with limited data and limited generalizability |
| 0-2 hours | 39.12 | 28.24 | 54.18 | 2 | 1 | 1 | Very Low | Sparse mixed-design data with limited precision and limited generalizability |
| 3-4 hours | NA | NA | NA | 0 | 0 | 0 | NA | No contributing cohorts |
| 6-8 hours | 0.64 | 0.53 | 0.76 | 2 | 2 | 0 | Low | Two RCT-derived cohorts with consistent estimates but limited data |
| 12 hours | NA | NA | NA | 0 | 0 | 0 | NA | No contributing cohorts |
| 18-20 hours | NA | NA | NA | 0 | 0 | 0 | NA | No contributing cohorts |
| POD 1 | 5.54 | 2.88 | 10.66 | 8 | 5 | 3 | Low | Mixed study designs showing postoperative CRP elevation; downgraded for limited cohort number and heterogeneity |
| POD 2 | 12.31 | 5.44 | 27.88 | 4 | 2 | 2 | Low | Few mixed-design cohorts with limited precision and generalizability |
| POD 3 | 5.17 | 5.1 | 5.24 | 3 | 0 | 3 | Very Low | Observational-only evidence from few cohorts despite consistent values |

**Table 8.** GRADE summary of certainty in the evidence for interleukin-8 (IL-8) trajectories following pediatric cardiopulmonary bypass.

| **Time Point** | **Pooled Estimate (pg/ml)** | **Lower CI** | **Upper CI** | **Number of Cohorts** | **RCTs** | **Observational Studies** | **GRADE Rating** | **Rationale** |
| --- | --- | --- | --- | --- | --- | --- | --- | --- |
| Baseline | 8.54 | 6.09 | 11.99 | 31 | 8 | 11 | Moderate | Multiple contributing cohorts with relatively precise baseline estimate; downgraded for mixed study designs and assay/population heterogeneity |
| Post CPB | 37.17 | 18.97 | 72.86 | 13 | 9 | 4 | Moderate | Predominantly RCT-derived cohorts showing early postoperative IL-8 elevation; downgraded for heterogeneity and imprecision |
| 0-2 hours | 54.39 | 27.13 | 109.06 | 7 | 3 | 4 | Low | Mixed RCT and observational cohorts with plausible early postoperative elevation; downgraded for imprecision, heterogeneity, and limited cohort number |
| 3-4 hours | 113.59 | 34.09 | 378.44 | 4 | 2 | 2 | Very Low | Few mixed-design cohorts with high variability and wide confidence interval |
| 6-8 hours | 48.86 | 25.37 | 94.09 | 7 | 5 | 2 | Moderate | Predominantly RCT-derived cohorts with coherent postoperative elevation; downgraded for expected clinical and methodological heterogeneity |
| 12 hours | 38.77 | 26.98 | 55.72 | 6 | 3 | 3 | Low | Limited number of mixed-design cohorts; downgraded for sparse data and heterogeneity |
| 18-20 hours | 81.12 | 74.59 | 88.22 | 1 | 1 | 0 | Very Low | Single RCT-derived estimate with limited generalizability |
| POD 1 | 21.32 | 12.25 | 37.13 | 14 | 8 | 6 | Moderate | Multiple cohorts, mostly RCT-derived, showing declining but persistent postoperative elevation; downgraded for heterogeneity |
| POD 2 | 30.72 | 18.62 | 50.68 | 3 | 1 | 2 | Low | Few cohorts and predominantly observational evidence; limited precision and generalizability |

**Table 9.** GRADE summary of certainty in the evidence for tumor necrosis factor-alpha (TNF-α) trajectories following pediatric cardiopulmonary bypass.

| **Time Point** | **Pooled Estimate (pg/ml)** | **Lower CI** | **Upper CI** | **Number of Cohorts** | **RCTs** | **Observational Studies** | **GRADE Rating** | **Rationale** |
| --- | --- | --- | --- | --- | --- | --- | --- | --- |
| Baseline | 91.05 | 23.33 | 355.3 | 21 | 13 | 8 | Low | Predominantly RCT-derived cohorts, but downgraded for very wide confidence interval and substantial baseline heterogeneity |
| Post CPB | 258.59 | 24.26 | 2756.61 | 12 | 8 | 4 | Very Low | Very wide confidence interval with substantial heterogeneity and limited confidence in the absolute pooled estimate |
| 0-2 hours | 13.38 | 10.04 | 17.83 | 2 | 1 | 1 | Very Low | Sparse mixed-design data with limited generalizability |
| 3-4 hours | 17.38 | 8.2 | 36.86 | 2 | 1 | 1 | Very Low | Sparse mixed-design data with limited precision and generalizability |
| 6-8 hours | 26.19 | 10.2 | 67.23 | 7 | 6 | 1 | Low | Predominantly RCT-derived cohorts, but downgraded for wide confidence interval and heterogeneity |
| 12 hours | 8.23 | 5.62 | 12.05 | 2 | 2 | 0 | Low | Two RCT-derived cohorts with relatively precise estimate, but limited data and generalizability |
| 18-20 hours | 50.88 | 6.17 | 419.74 | 3 | 3 | 0 | Very Low | Few RCT-derived cohorts with wide confidence interval and limited generalizability |
| POD 1 | 66.79 | 11.86 | 376.07 | 14 | 9 | 5 | Low | Predominantly RCT-derived and mixed-design cohorts, but downgraded for wide confidence interval and substantial heterogeneity |
| POD 2 | 251.2 | 3.61 | 17468.46 | 3 | 2 | 1 | Very Low | Few cohorts with extremely wide confidence interval and possible outlier influence |
| POD 3 | 4674.41 | 1.31 | 16691415.12 | 2 | 1 | 1 | Very Low | Sparse mixed-design data with implausibly wide confidence interval and very low reliability of the pooled estimate |

**Figure 1.** Overview of GRADE certainty of evidence across postoperative time points for key cytokines.

1.	Keski-Nisula J, Arvola O, Jahnukainen T, Andersson S, Pesonen E. Reduction of Inflammation by High-Dose Methylprednisolone Does not Attenuate Oxidative Stress in Children Undergoing Bidirectional Glenn Procedure With or Without Aortic Arch or Pulmonary Arterial Repair. *J Cardiothorac Vasc Anesth* 2020; **34**(6): 1542-7.

2.	Ming ZD, Wei W, Hong C, Wei Z, Xiang DW. Balanced ultrafiltration, modified ultrafiltration, and balanced ultrafiltration with modified ultrafiltration in pediatric cardiopulmonary bypass. *J Extra Corpor Technol* 2001; **33**(4): 223-6.

3.	Gu CH, Cui Q, Wang YY, et al. Effects of insulin therapy on inflammatory mediators in infants undergoing cardiac surgery with cardiopulmonary bypass. *Cytokine* 2008; **44**(1): 96-100.

4.	Inoue N, Oka N, Kitamura T, et al. Neutrophil elastase inhibitor sivelestat attenuates perioperative inflammatory response in pediatric heart surgery with cardiopulmonary bypass. *Int Heart J* 2013; **54**(3): 149-53.

5.	Kaskinen AK, Keski-Nisula J, Martelius L, et al. Lung Injury After Neonatal Congenital Cardiac Surgery Is Mild and Modifiable by Corticosteroids. *J Cardiothorac Vasc Anesth* 2021; **35**(7): 2100-7.

6.	Gessler P, Pretre R, Hohl V, Rousson V, Fischer J, Dahinden C. CXC-chemokine stimulation of neutrophils correlates with plasma levels of myeloperoxidase and lactoferrin and contributes to clinical outcome after pediatric cardiac surgery. *Shock* 2004; **22**(6): 513-20.

7.	Appachi E, Mossad E, Mee RB, Bokesch P. Perioperative serum interleukins in neonates with hypoplastic left-heart syndrome and transposition of the great arteries. *J Cardiothorac Vasc Anesth* 2007; **21**(2): 184-90.

8.	Gorjipour F, Dehaki MG, Totonchi Z, et al. Inflammatory cytokine response and cardiac troponin I changes in cardiopulmonary bypass using two cardioplegia solutions; del Nido and modified St. Thomas': a randomized controlled trial. *Perfusion* 2017; **32**(5): 394-402.

9.	Cheung MM, Kharbanda RK, Konstantinov IE, et al. Randomized controlled trial of the effects of remote ischemic preconditioning on children undergoing cardiac surgery: first clinical application in humans. *J Am Coll Cardiol* 2006; **47**(11): 2277-82.

10.	Draaisma AM, Hazekamp MG, Anes N, et al. Phosphorylcholine coating of bypass systems used for young infants does not attenuate the inflammatory response. *Ann Thorac Surg* 2006; **81**(4): 1455-9.

11.	Xie Y, Jiang W, Cao J, Xie H. Dexmedetomidine attenuates acute kidney injury in children undergoing congenital heart surgery with cardiopulmonary bypass by inhibiting the TLR3/NF-κB signaling pathway. *Am J Transl Res* 2021; **13**(4): 2763-73.

12.	Caputo M, Bays S, Rogers CA, et al. Randomized comparison between normothermic and hypothermic cardiopulmonary bypass in pediatric open-heart surgery. *Ann Thorac Surg* 2005; **80**(3): 982-8.

13.	Alcaraz AJ, Sancho L, Manzano L, et al. Newborn patients exhibit an unusual pattern of interleukin 10 and interferon gamma serum levels in response to cardiac surgery. *J Thorac Cardiovasc Surg* 2002; **123**(3): 451-8.

14.	Berdat PA, Eichenberger E, Ebell J, et al. Elimination of proinflammatory cytokines in pediatric cardiac surgery: analysis of ultrafiltration method and filter type. *J Thorac Cardiovasc Surg* 2004; **127**(6): 1688-96.

15.	Gessler P, Pretre R, Bürki C, Rousson V, Frey B, Nadal D. Monocyte function-associated antigen expression during and after pediatric cardiac surgery. *J Thorac Cardiovasc Surg* 2005; **130**(1): 54-60.

16.	Joashi U, Tibby SM, Turner C, et al. Soluble Fas may be a proinflammatory marker after cardiopulmonary bypass in children. *J Thorac Cardiovasc Surg* 2002; **123**(1): 137-44.

17.	Sason-Ton Y, Ben Abraham R, Lotan D, et al. Tumor necrosis factor and clinical and metabolic courses after cardiac surgery in children. *J Thorac Cardiovasc Surg* 2002; **124**(5): 991-8.

18.	Arkader R, Troster EJ, Abellan DM, et al. Procalcitonin and C-reactive protein kinetics in postoperative pediatric cardiac surgical patients. *J Cardiothorac Vasc Anesth* 2004; **18**(2): 160-5.

19.	Bulutcu FS, Bayindir O, Polat B, Yalcin Y, öZbek U, Cakali E. Does normoxemic cardiopulmonary bypass prevent myocardial reoxygenation injury in cyanotic children? *J Cardiothorac Vasc Anesth* 2002; **16**(3): 330-3.

20.	Christen S, Finckh B, Lykkesfeldt J, et al. Oxidative stress precedes peak systemic inflammatory response in pediatric patients undergoing cardiopulmonary bypass operation. *Free Radic Biol Med* 2005; **38**(10): 1323-32.

21.	Hammer S, Fuchs AT, Rinker C, Daebritz S, Kozlik-Feldmann R, Netz H. Interleukin-6 and procalcitonin in serum of children undergoing cardiac surgery with cardiopulmonary bypass. *Acta Cardiol* 2004; **59**(6): 624-9.

22.	Bhutta AT, Schmitz ML, Swearingen C, et al. Ketamine as a neuroprotective and anti-inflammatory agent in children undergoing surgery on cardiopulmonary bypass: a pilot randomized, double-blind, placebo-controlled trial. *Pediatr Crit Care Med* 2012; **13**(3): 328-37.

23.	Tassani P, Schad H, Schreiber C, et al. Extravasation of albumin after cardiopulmonary bypass in newborns. *J Cardiothorac Vasc Anesth* 2007; **21**(2): 174-8.

24.	Zhao J, Liu JP, Feng ZY, Liu YL, Li SJ, Long C. Clinical application of pulsatile perfusion during cardiopulmonary bypass in pediatric heart surgery. *Asaio j* 2009; **55**(3): 300-3.

25.	Wang W, Zhu DM, Huang HM, et al. Effect of flow rate, negative pressure, and duration of modified ultrafiltration on hemodynamics and inflammatory mediators. *ASAIO J* 2007; **53**(1): 41-5.

26.	Nasser BA, Mesned AR, Tageldein M, Kabbani MS, Sayed NS. Can acute-phase response biomarkers differentiate infection from inflammation postpediatric cardiac surgery? *Avicenna J Med* 2017; **7**(4): 182-8.

27.	Mikłaszewska M, Korohoda P, Zachwieja K, et al. Serum interleukin 6 levels as an early marker of acute kidney injury on children after cardiac surgery. *Adv Clin Exp Med* 2013; **22**(3): 377-86.

28.	Madhok AB, Ojamaa K, Haridas V, Parnell VA, Pahwa S, Chowdhury D. Cytokine response in children undergoing surgery for congenital heart disease. *Pediatr Cardiol* 2006; **27**(4): 408-13.

29.	Chasovskyi K, Zhovnir V, Vorobiova G, Yemets I. Relationship Between Antiinflammatory Cytokine Interleukin-10 and Lactate Concentrations in Neonates Undergoing the Arterial Switch Operation. *Ann Thorac Surg* 2016; **101**(3): 1020-4.

30.	Kesumarini D, Widyastuti Y, Boom CE, Dinarti LK. Effectiveness of Dexmedetomidine as Myocardial Protector in Children With Classic Tetralogy of Fallot Having Corrective Surgery: A Randomized Controlled Trial. *J Cardiothorac Vasc Anesth* 2024; **38**(6): 1369-77.

31.	Lang SM, Syed MA, Dziura J, et al. The effect of modified ultrafiltration on angiopoietins in pediatric cardiothoracic operations. *Ann Thorac Surg* 2014; **98**(5): 1699-704.

32.	Liu X, Chen Q, Luo Y, et al. Plasma levels of alarmin HNPs 1-3 associate with lung dysfunction after cardiac surgery in children. *BMC Pulm Med* 2017; **17**(1): 218.

33.	Liu KD, Altmann C, Smits G, et al. Serum interleukin-6 and interleukin-8 are early biomarkers of acute kidney injury and predict prolonged mechanical ventilation in children undergoing cardiac surgery: a case-control study. *Crit Care* 2009; **13**(4): R104.

34.	Greenberg JH, Whitlock R, Zhang WR, et al. Interleukin-6 and interleukin-10 as acute kidney injury biomarkers in pediatric cardiac surgery. *Pediatr Nephrol* 2015; **30**(9): 1519-27.

35.	Fakhri D, Marwali EM, Budiwardhana N, Roebiono PS, Rahajoe AU, Caesario M. Diagnosing infection after infant open heart surgery: role of procalcitonin. *Asian Cardiovasc Thorac Ann* 2019; **27**(8): 641-5.

36.	Aĝirbaşli M, Nguyen ML, Win K, et al. Inflammatory and hemostatic response to cardiopulmonary bypass in pediatric population: feasibility of seriological testing of multiple biomarkers. *Artif Organs* 2010; **34**(11): 987-95.

37.	Varan B, Tokel K, Mercan S, Dönmez A, Aslamaci S. Systemic inflammatory response related to cardiopulmonary bypass and its modification by methyl prednisolone: high dose versus low dose. *Pediatr Cardiol* 2002; **23**(4): 437-41.

38.	Talwar S, Bhoje A, Sreenivas V, et al. Comparison of del Nido and St Thomas Cardioplegia Solutions in Pediatric Patients: A Prospective Randomized Clinical Trial. *Semin Thorac Cardiovasc Surg* 2017; **29**(3): 366-74.

39.	Sever K, Tansel T, Basaran M, et al. The benefits of continuous ultrafiltration in pediatric cardiac surgery. *Scand Cardiovasc J* 2004; **38**(5): 307-11.

40.	Schindler E, Photiadis J, Lagudka S, Fink C, Hraska V, Asfour B. Influence of two perfusion strategies on oxygen metabolism in paediatric cardiac surgery. Evaluation of the high-flow, low-resistance technique. *Eur J Cardiothorac Surg* 2010; **37**(3): 651-7.

41.	Reiter K, Balling G, Bonelli V, et al. Neutrophil gelatinase-associated lipocalin reflects inflammation and is not a reliable renal biomarker in neonates and infants after cardiopulmonary bypass: a prospective case-control study. *Cardiol Young* 2018; **28**(2): 243-51.

42.	Poyrazoğlu HH, Duman Z, Demir Ş, et al. Investigating the Impacts of Preoperative Steroid Treatment on Tumor Necrosis Factor-Alpha and Duration of Extubation Time underwent Ventricular Septal Defect Surgery. *Balkan Med J* 2016; **33**(2): 158-63.

43.	Pourmoghadam KK, Kubovec S, DeCampli WM, et al. Passive Peritoneal Drainage Impact on Fluid Balance and Inflammatory Mediators: A Randomized Pilot Study. *World J Pediatr Congenit Heart Surg* 2020; **11**(2): 150-8.

44.	McMahon CK, Klein I, Ojamaa K. Interleukin-6 and thyroid hormone metabolism in pediatric cardiac surgery patients. *Thyroid* 2003; **13**(3): 301-4.

45.	Olsson C, Siegbahn A, Henze A, et al. Heparin-coated cardiopulmonary bypass circuits reduce circulating complement factors and interleukin-6 in paediatric heart surgery. *Scand Cardiovasc J* 2000; **34**(1): 33-40.

46.	Sethi BS, Kapoor PM, Chauhan S, Chowdhury UK, Kiran U, Choudhury M. Perioperative levels of tumor necrosis factor-α correlate with outcomes in children and adults with tetralogy of Fallot undergoing corrective surgery. *World J Pediatr Congenit Heart Surg* 2014; **5**(1): 38-46.

47.	Osthaus WA, Görler H, Sievers J, et al. Bicarbonate-buffered ultrafiltration during pediatric cardiac surgery prevents electrolyte and acid-base balance disturbances. *Perfusion* 2009; **24**(1): 19-25.

48.	Umstead TM, Lu CJ, Freeman WM, et al. Dual-platform proteomics study of plasma biomarkers in pediatric patients undergoing cardiopulmonary bypass. *Pediatr Res* 2010; **67**(6): 641-9.

49.	Merchant S, Nadaraj S, Chowdhury D, et al. Macrophage migration inhibitory factor in pediatric patients undergoing surgery for congenital heart repair. *Mol Med* 2008; **14**(3-4): 124-30.

50.	Rhodes LA, Robert SM, Atkinson TP, Dabal RJ, Mahdi AM, Alten JA. Hypogammaglobulinemia after cardiopulmonary bypass in infants. *J Thorac Cardiovasc Surg* 2014; **147**(5): 1587-93.e1.

51.	McQuinn TC, Deardorff RL, Mukherjee R, et al. Circulating matrix metalloproteinase levels after ventricular septal defect repair in infants. *J Thorac Cardiovasc Surg* 2010; **140**(6): 1257-65.

52.	Walavalkar V, Evers E, Pujar S, et al. Preoperative Sildenafil administration in children undergoing cardiac surgery: a randomized controlled preconditioning study. *Eur J Cardiothorac Surg* 2016; **49**(5): 1403-10.

53.	Verdesoto Rodriguez MC, Spenceley N, Ilina M, Danton MHD. A Prospective Randomized Blinded Trial of Remote Ischemic Preconditioning in Children Undergoing Cardiac Surgery. *Semin Thorac Cardiovasc Surg* 2020; **32**(2): 313-22.

54.	Wheeler DS, Bigham MT, Lahni PM, et al. The extracellular stress response to pediatric cardiopulmonary bypass. *J Pediatr Intensive Care* 2014; **3**(1): 9-16.

55.	Zhou W, Zeng D, Chen R, et al. Limb ischemic preconditioning reduces heart and lung injury after an open heart operation in infants. *Pediatr Cardiol* 2010; **31**(1): 22-9.

56.	Grosek S, Primozic J, Ihan A, Wraber B, Gabrijelcic T, Kosin M. Interleukin-10, T-lymphocytes, and cardiac output in children after ventricular septal defect repair: a pilot study. *Intensive Care Med* 2006; **32**(5): 780-4.

57.	Nuszkowski MM, Deutsch N, Jonas RA, Zurakowski D, Montague E, Holt DW. Randomized trial of the Terumo Capiox FX05 oxygenator with integral arterial filter versus Terumo Capiox Baby RX05 and Terumo Capiox AF02 arterial filter in infants undergoing cardiopulmonary bypass. *J Extra Corpor Technol* 2011; **43**(4): 207-14.

58.	Kagawa H, Morita K, Uno Y, et al. Inflammatory response to hyperoxemic and normoxemic cardiopulmonary bypass in acyanotic pediatric patients. *World J Pediatr Congenit Heart Surg* 2014; **5**(4): 541-5.

59.	Ibrahim TH, Abdelrahman HS, Alharbi MA, Zabani IA, Ismail MF, Kary H. Effect of ketamine on pro- and anti-inflammatory cytokine response in paediatric cardiac surgery: A prospective randomised controlled study. *Indian J Anaesth* 2017; **61**(7): 549-55.

60.	Gessler P, Hohl V, Carrel T, et al. Administration of steroids in pediatric cardiac surgery: impact on clinical outcome and systemic inflammatory response. *Pediatr Cardiol* 2005; **26**(5): 595-600.

61.	Fleming GA, Billings FTt, Klein TM, Bichell DP, Christian KG, Pretorius M. Angiotensin-converting enzyme inhibition alters the inflammatory and fibrinolytic response to cardiopulmonary bypass in children. *Pediatr Crit Care Med* 2011; **12**(5): 532-8.

62.	Cholette JM, Henrichs KF, Alfieris GM, et al. Washing red blood cells and platelets transfused in cardiac surgery reduces postoperative inflammation and number of transfusions: results of a prospective, randomized, controlled clinical trial. *Pediatr Crit Care Med* 2012; **13**(3): 290-9.

63.	Caputo M, Mokhtari A, Rogers CA, et al. The effects of normoxic versus hyperoxic cardiopulmonary bypass on oxidative stress and inflammatory response in cyanotic pediatric patients undergoing open cardiac surgery: a randomized controlled trial. *J Thorac Cardiovasc Surg* 2009; **138**(1): 206-14.

64.	Amanullah MM, Hamid M, Hanif HM, et al. Effect of steroids on inflammatory markers and clinical parameters in congenital open heart surgery: a randomised controlled trial. *Cardiol Young* 2016; **26**(3): 506-15.

65.	Dhillon S, Yu X, Cheypesh A, Ross DB, Li J. Comparison of Profiles of Perioperative Serum C-Reactive Protein Levels in Neonates Undergoing the Norwood Procedure or Arterial Switch Operation. *Congenit Heart Dis* 2015; **10**(3): 226-33.

66.	Davidson J, Tong S, Hauck A, et al. Alkaline phosphatase activity after cardiothoracic surgery in infants and correlation with post-operative support and inflammation: a prospective cohort study. *Crit Care* 2012; **16**(4): R160.

67.	Chew MS, Brandslund I, Brix-Christensen V, et al. Tissue injury and the inflammatory response to pediatric cardiac surgery with cardiopulmonary bypass: a descriptive study. *Anesthesiology* 2001; **94**(5): 745-53; discussion 5A.

68.	Ben-Abraham R, Weinbroum AA, Lotan D, et al. Interleukin-8 secretion following cardiopulmonary bypass in children as a marker of early postoperative morbidity. *Paediatr Anaesth* 2002; **12**(2): 156-61.

69.	Baek HS, Lee Y, Jang HM, et al. Variation in clinical usefulness of biomarkers of acute kidney injury in young children undergoing cardiac surgery. *Clin Exp Pediatr* 2020; **63**(4): 151-6.

70.	Alcaraz AJ, Manzano L, Sancho L, et al. Different proinflammatory cytokine serum pattern in neonate patients undergoing open heart surgery. Relevance of IL-8. *J Clin Immunol* 2005; **25**(3): 238-45.

71.	Jahnukainen T, Keski-Nisula J, Tainio J, et al. Efficacy of corticosteroids in prevention of acute kidney injury in neonates undergoing cardiac surgery-A randomized controlled trial. *Acta Anaesthesiol Scand* 2018; **62**(8): 1072-9.
